# Machine learning discovery of missing links that mediate alternative branches to plant alkaloids

**DOI:** 10.1038/s41467-022-28883-8

**Published:** 2022-03-16

**Authors:** Christopher J. Vavricka, Shunsuke Takahashi, Naoki Watanabe, Musashi Takenaka, Mami Matsuda, Takanobu Yoshida, Ryo Suzuki, Hiromasa Kiyota, Jianyong Li, Hiromichi Minami, Jun Ishii, Kenji Tsuge, Michihiro Araki, Akihiko Kondo, Tomohisa Hasunuma

**Affiliations:** 1 Graduate School of Science, Technology and Innovation, Kobe University, 1-1 Rokkodai, Nada-ku, Kobe, 657-8501 Japan; 2 Division of Life Science, School of Science and Engineering, Tokyo Denki University, Hatoyama, Hiki-gun, Saitama, 350-0394 Japan; 3 Department of Chemical Science and Engineering, Graduate School of Engineering, Kobe University, 1-1 Rokkodai, Nada-ku, Kobe, 657-8501 Japan; 4 Faculty of Agriculture, Okayama University, 1-1-1, Tsushima-Naka, Kita-ku, Okayama, 700-8530 Japan; 5 Department of Biochemistry, Virginia Polytechnic and State University, 111 Engel Hall, Mail Code: 0308, Blacksburg, VA 24061 USA; 6 Research Institute for Bioresources and Biotechnology, Ishikawa Prefectural University, 1-308, Suematsu, Nonoichi-shi, Ishikawa-ken, 921-8836 Japan; 7 Engineering Biology Research Center, Kobe University, 1-1 Rokkodai, Nada-ku, Kobe, 657-8501 Japan; 8 Graduate School of Medicine, Kyoto University, Yoshida-Konoe-cho, Sakyo-ku, Kyoto, 606-8501 Japan; 9 National Institutes of Biomedical Innovation, Health and Nutrition, 1-23-1 Toyama, Shinjuku-ku, Tokyo, 162-8638 Japan

**Keywords:** Enzymes, Synthetic biology, Metabolic engineering, Machine learning

## Abstract

Engineering the microbial production of secondary metabolites is limited by the known reactions of correctly annotated enzymes. Therefore, the machine learning discovery of specialized enzymes offers great potential to expand the range of biosynthesis pathways. Benzylisoquinoline alkaloid production is a model example of metabolic engineering with potential to revolutionize the paradigm of sustainable biomanufacturing. Existing bacterial studies utilize a norlaudanosoline pathway, whereas plants contain a more stable norcoclaurine pathway, which is exploited in yeast. However, committed aromatic precursors are still produced using microbial enzymes that remain elusive in plants, and additional downstream missing links remain hidden within highly duplicated plant gene families. In the current study, machine learning is applied to predict and select plant missing link enzymes from homologous candidate sequences. Metabolomics-based characterization of the selected sequences reveals potential aromatic acetaldehyde synthases and phenylpyruvate decarboxylases in reconstructed plant gene-only benzylisoquinoline alkaloid pathways from tyrosine. Synergistic application of the aryl acetaldehyde producing enzymes results in enhanced benzylisoquinoline alkaloid production through hybrid norcoclaurine and norlaudanosoline pathways.

## Abstract (web summary)

Producing plant secondary metabolites by microbes is limited by the known enzymatic reactions. Here, the authors apply machine learning to predict missing link enzymes of benzylisoquinoline alkaloid (BIA) biosynthesis in *Papaver somniferum*, and validate the specialized activities through heterologous production.

## Introduction

Strain engineering is now a reliable approach to scale up the production of target metabolites by integrating known genes, and applying simple yet effective metabolic engineering strategies^1^. But engineering the microbial production of secondary metabolites reaches the limitation of the characterized enzymes present in sequence databases, where many annotations are incorrect. In reality, there are millions of enzyme variants to choose from for each desired reaction, and a great abundance of variations are still hidden in nature with unknown sequence and function. In this way, the evolution of nature over millions of years can be viewed as a highly diverse screening resource for synthetic biologists. Accordingly, the rational discovery of homologous enzyme sequences with useful functions is a powerful and inevitable approach to improve microbial bioproduction pathways^2–6^.

Functional prediction of uncharacterized enzyme sequences is a promising approach to increase the number of specialized enzymes that can be applied to biosynthesis^5,7,8^. In our previous study, aromatic acetaldehyde synthase (AAS) was predicted with the enzyme selection software M-path to improve the production of valuable alkaloids^7^. However, only enzyme commission (EC) number could be predicted with M-path and the actual selection of candidate sequences had to be performed by human intuition. To address this issue, a support vector machine (SVM) algorithm was developed to automatically select specific enzyme sequences:^8^ an upgrade that enables computer automated Design, Build, Test and Learn (DBTL) cycles.

**Fig. 1:**
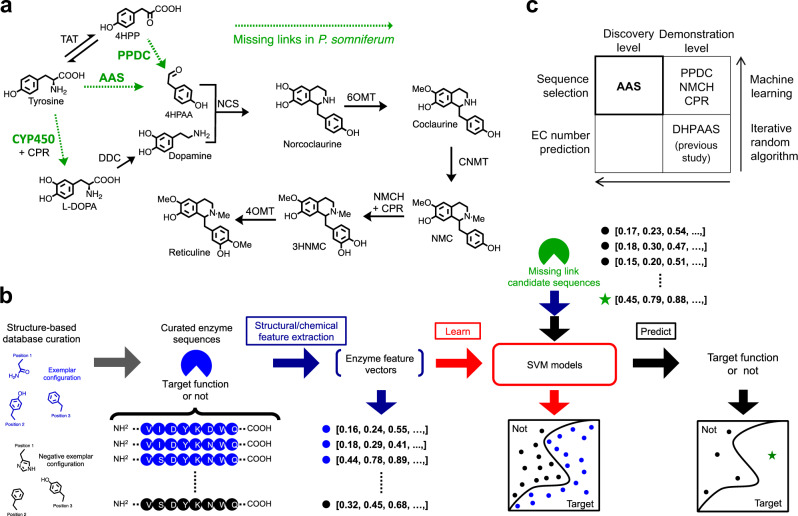
Uncovering missing links in *Papaver somniferum* as alternative branches to benzylisoquinoline alkaloids. **a** Steps mediated by unclear *P. somniferum* enzymes are shown as green dotted arrows. Steps with black arrows are added to the *E. coli* chassis by plasmid transformation, except TAT^49^ which is naturally present in *E. coli* as aromatic-amino-acid aminotransferase (tyrB). Metabolite abbreviations: 4HPP 4-hydroxyphenylpyruvic acid, 4HPAA 4-hydroxyphenylacetaldehyde, L-DOPA 3,4-dihydroxy-L-phenylalanine, DHPAA 3,4-dihydroxyphenylacetaldehyde, NMC *N*-methylcoclaurine, 3HNMC 3-hydroxy-*N*-methylcoclaurine. Enzyme abbreviations: AAS aromatic acetaldehyde synthase, DHPAAS 3,4-dihydroxyphenylacetaldehyde synthase, PPDC phenylpyruvate decarboxylase, DDC L-DOPA decarboxylase, CYP450 cytochrome P450, CPR CYP450 reductase, ARO10 *Saccharomyces cerevisiae* transaminated amino acid decarboxylase, NCS norcoclaurine synthase, 6OMT norcoclaurine 6-*O*-methyltransferase, CNMT coclaurine *N*-methyltransferase, NMCH *N*-methylcoclaurine 3-hydroxylase, 4OMT 3-hydroxy-*N*-methylcoclaurine 4-*O*-methyltransferase. **b** Missing link candidate sequences are predicted and ranked based on high-dimensional support vector machine (SVM) models^8^. Structure-based rules are first determined to curate training sequences as those of the target function (blue) and to differentiate related sequences with a different function (black). Structural and chemical features are then extracted from training and test sequences^54, 57^, resulting in enzyme feature vectors. SVM models are built with the training vectors^8^, and vectors from missing link candidate sequences are then tested against the models to predict their specialized function. **c** Four quadrants emerge when classifying enzyme prediction based on Enzyme Commission (EC) number prediction versus sequence selection, and demonstration level versus discovery level.

Conversion of tyrosine to benzylisoquinoline alkaloid (BIA) is a model example of metabolic engineering (Fig. 1)^1,4,5,7^. BIAs are precursors to opioid analgesic medications that are currently mass-produced by industrially grown *Papaver somniferum* plants, which are a historical target for the human-directed evolution of natural product production. While opioid misuse is a global problem, natural and semi-synthetic opioids derived from the BIA reticuline actually result in fewer deaths than less expensive and overly potent synthetic opioids (CDC Opioid Data Analysis and Resources). With diverse potential, natural BIAs have been shown to inhibit coronavirus^9^, and the BIA norcoclaurine is a β2-adrenergic receptor agonist that is present in edible plants, medicinal herbs and sports supplements^10,11^.

BIA production in *Escherichia coli* has utilized bacterial monoamine oxidase and insect 3,4-dihydroxyphenylacetaldehyde synthase (DHPAAS) to generate toxic 3,4-dihydroxyphenylacetaldehyde (DHPAA)^12^. However, the DHPAA containing pathways result in rapid loss of unstable catechol containing intermediates^7,12–15^. Other reports show that plants use a 4-hydroxyphenylacetaldehyde (4HPAA) pathway to norcoclaurine (Supplementary Fig. 1), which may be more stable due to lack of a catechol group in early intermediates. Therefore, plant 4HPAA pathways offer the potential to prevent the loss of BIA intermediates in *E. coli*. Furthermore, the combination of 4HPAA and DHPAA pathways may also improve the utilization of tyrosine and aryl acetaldehydes. Despite success with the 4HPAA pathway in yeast^1,4,16,17^ and many discussions on the expected phenylpyruvate decarboxylase (PPDC, EC 4.1.1.43) and AAS (EC 4.1.107-9)^18–20^ activities in plants, no enzymes to produce aryl acetaldehydes 4HPAA or DHPAA have been characterized from high alkaloid producing poppy plants^21^. Moreover, no plant sequence annotated as phenylpyruvate decarboxylase can be found from public databases, and numerous *P. somniferum* cytochrome P450 (CYP450) monooxygenases (EC 1.14.14) require complex clarification. Therefore, this serious limitation in known enzymes is addressed by applying machine learning to predict the specialized missing links in plant alkaloid pathways shown as dotted arrows in Fig. 1 and Supplementary Fig. 1.

In this study, eight refined SVM models are built and applied to automate the selection of target sequences from over 100 candidates present throughout highly duplicated carboxy-lyase and oxidase gene families. Then, to verify the machine learning predictions, approximately 50 strains expressing various combinations of candidate sequences are screened using liquid chromatography-mass spectrometry (LC-MS)-, capillary electrophoresis–MS (CE-MS)- and gas chromatography–MS (GC-MS)-based metabolomics. As a result, AAS and PPDC are identified as potential missing links that mediate uncharacterized branches of the *Papaver somniferum* alkaloid pathway. The synergistic combination of predicted enzymes together with homologous enzyme templates affords 356 µM norcoclaurine, 240 µM *N*-methylcoclaurine and 74.9 µM reticuline, without using any genome engineering. The alternative branches of flux from tyrosine to downstream alkaloids are confirmed using dynamic metabolic profiling^5^ with mechanism-directed deuterium labeling patterns.

## Results

### Prediction and discovery of *P. somniferum* aromatic acetaldehyde synthase

**Fig. 2:**
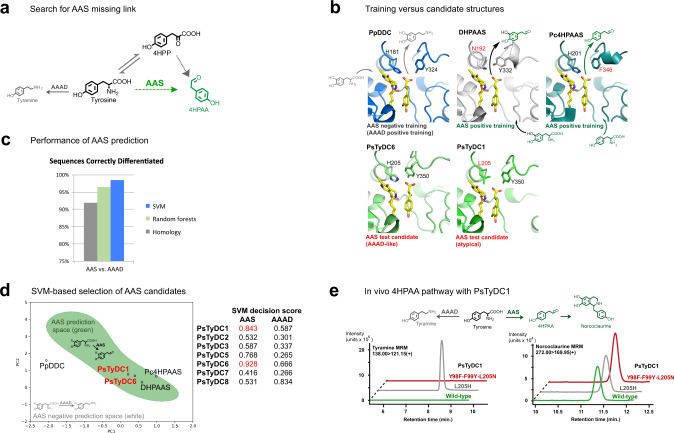
Prediction of AAS branch pathway enzymes to produce 4HPAA for norcoclaurine production. **a** The aromatic acetaldehyde synthase (AAS) branch pathway (green) can produce 4HPAA directly from tyrosine, but this missing link is unreported in *P. somniferum*. **b** Structure-based curation of typical aromatic amino acid decarboxylase (AAAD), insect-type AAS and plant-type AAS, as represented by the active site configurations of *Pseudomonas putida* DDC (PpDDC, blue), *Bombyx mori* DHPAAS (DHPAAS, grey), and *Petroselinum crispum* 4HPAAS (Pc4HPAAS, deepteal). AAS candidate PsTyDC1 (green) has a unique active site, while AAS candidate PsTyDC6 (green) has an AAAD-like active site and could not be predicted by a homology or structure-based approach alone. **c** Cross-validation for correct assignment of AAAD and AAS training sequences is performed using SVM models (blue), Random forests models (green), and by comparing sequence homology of each training sequence to a consensus sequence of AAS training sequences and a consensus sequence of AAAD training sequences (grey), as described in the methods section. **d** For visual representation, a two-dimensional plot of AAS SVM-based prediction is shown, with positive and negative prediction spaces colored green and white, respectively (left side). Principal component analysis (PCA) is used to compress multi-dimensional data into two dimensions (PC1 and PC2) for a visual representation. Corresponding high-dimensional SVM decision scores from Supplementary Table 1 are shown on the right. Decision scores represent the distance from the SVM prediction boundary. PsTyDC1 and PsTyDC6 score highest for AAS prediction and are colored red. **e** LC-MS detection of products from *Thalictrum flavum* norcoclaurine synthase (TfNCS) containing strains T1-01-DE3 (wild-type PsTyDC1 + TfNCS), T1-02-DE3 (PsTyDC1-L205H + TfNCS) and T1-03-DE3 (PsTyDC1-Y98F-F99Y-L205N + TfNCS) (Supplementary Table 2), grown in LB supplemented with 1 mM tyrosine and 0.5 mM dopamine, at 28 °C with 180 rpm shaking for 51 h. Selective in vivo production of the downstream AAS product norcoclaurine accompanies the expression of wild-type PsTyDC1 (green), as well as the triple variant of PsTyDC1 with an engineered active site based on that of insect DHPAAS (red). Tyramine is the major product of PsTyDC1-L205H (grey), which contains an engineered active site based on typical AAAD. Similar results are replicated in Supplementary Fig. 2 and Supplementary Fig. 3.

DHPAA and norlaudanosoline (also referred to as tetrahydropapaveroline or THP) are more easily oxidized and more toxic than their corresponding 4-hydroxyphenyl analogues^12^. Therefore, missing link enzymes to 4HPAA and norcoclaurine are explored to test our machine learning enzyme selection models (Fig. 2). Our previous M-path analysis identified 4-hydroxyphenylacetaldehyde synthase (4HPAAS, EC 4.1.1.108) to mediate 4HPAA production from tyrosine; however specific 4HPAAS sequences are incompletely annotated throughout databases. In this study the term AAS is used to cover plant-type AAS enzymes 4HPAAS and phenylacetaldehyde synthase (PAAS, EC 4.1.1.109), as well as insect 3,4-dihydroxyphenylacetaldehyde synthase (DHPAAS, EC 4.1.1.107), because substrate specificities are often mixed throughout these groups.

Unclear variations within the plant-type AAS group, which may act upon a wide range of substrates including phenylalanine, tyrosine, 3,4-dihydroxy-L-phenylalanine (L-DOPA), tryptophan, and histidine, further complicates the selection of a correct sequence based on phylogenetic and structural analyses alone. Accordingly, no AAS enzyme from *P. somniferum* has been clearly established^21^. To overcome this challenge in enzyme prediction, our SVM-based algorithm^8^ is first applied to select AAS from *P. somniferum* homologs annotated as tyrosine/DOPA decarboxylase (TyDC) (Fig. 2c, d, and Supplementary Table 1).

Separate SVM models for pyridoxal 5-phosphate (PLP)-dependent aromatic amino acid decarboxylase (AAAD) and closely related PLP-dependent AAS were trained using sequences classified as described in the methods section (Supplementary Data 1 and Supplementary Data 2). According to database annotations and previous reports, *P. somniferum* TyDC (PsTyDC) proteins should be expected to catalyze the decarboxylation of tyrosine to form tyramine, and possibly L-DOPA conversion to dopamine^22,23^. In contrast, SVM decision scores show that while most of the full-length PsTyDC sequences have high potential for AAAD activity, PsTyDC1-8 also appear in AAS prediction space (Fig. 2d and Supplementary Table 1). Here, higher positive SVM decision scores indicate sequences that are further from the SVM prediction boundary, deeper within the positive prediction space.

PsTyDC1 contains the unique active site residue L205 (Fig. 2b), further suggesting atypical activity of this test sequence, and PsTyDC1 is therefore first selected to explore demonstration level (Fig. 1c) prediction of AAS. In accordance with the SVM prediction, expression of wild-type PsTyDC1 in *E. coli* promotes in vivo production of norcoclaurine from tyrosine and dopamine (Fig. 2e). As a positive AAS control, PsTyDC1-Y98F-F99Y-L205N with engineered active site residues transplanted from insect DHPAAS, also produces similar results to those of wild-type PsTyDC1. After substitution of PsTyDC1-L205 to a histidine residue, found in typical AAAD, the decarboxylation product tyramine increases dramatically (Fig. 2e and Supplementary Fig. 2). Production of norcoclaurine is further confirmed in strains expressing PsTyDC1 with additional variations in the alkaloid pathway (Supplementary Fig. 3 and Supplementary Table 2).

While norcoclaurine is detected in vivo with the expression of PsTyDC1, in vitro production of unstable 4HPAA by PsTyDC1 could not be detected, possibly due to low activity of PsTyDC1. Therefore, the SVM models are investigated further to select a better AAS candidate that might not be suggested by structural analysis. Despite containing AAAD-like active site residues Y98, F99, H205, Y350, and S372 (Fig. 2b), PsTyDC6 scores highest in the AAS prediction model (Fig. 2d and Supplementary Table 1). Therefore, PsTyDC6 is further selected for in vitro characterization. Interestingly, PsTyDC6 and PsTyDC1 share over 98% sequence identity, which is the highest sequence identity among the entire PsTyDC family, and PsTyDC6 is accordingly annotated as ‘tyrosine/DOPA decarboxylase 1-like’.

**Fig. 3:**
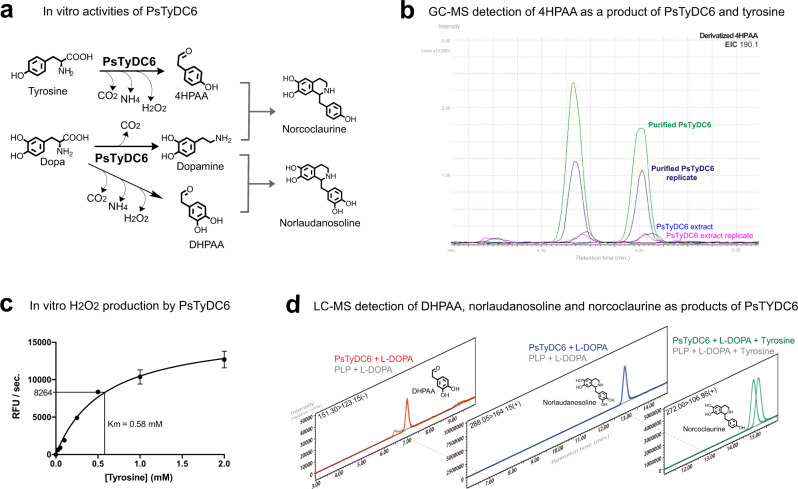
PsTyDC6 exhibits in vitro AAS activity. **a** PsTyDC6 produces 4HPAA and tyramine from tyrosine, and DHPAA and dopamine from L-DOPA. **b** Derivatized 4HPAA, from reactions with purified PsTyDC6 (green and dark blue) and PsTyDC6 extracts (blue and magenta), is detected with GC-MS, as shown in the extracted ion chromatogram (EIC). Lyophilized enzyme reactions were derivatized with methoxyamine in pyridine and MSTFA before analysis with GC-MS. **c** H_2_O_2_ production accompanies AAS activity. PsTyDC6 produced H_2_O_2_ in the presence of tyrosine as indicated by a peroxidase-based fluorescent assay. Error bars represent mean values +/− standard deviations, from three independent tests for each condition (*n* = 3). **d** DHPAA (red), as well as downstream products norlaudanosoline (blue) and norcoclaurine (green), are detected with LC-MS. All three products were not detected in control samples (grey), containing PLP cofactor in place of PsTyDC6. Source data are provided as a Source Data file.

In agreement with the high AAS decision score, PsTyDC6 exhibits AAS activity in the presence of tyrosine and L-DOPA (Fig. 3), thereby demonstrating discovery level (Fig. 1c) prediction of a plant AAS enzyme. Here, the in vitro AAS activity of PsTyDC6 is indicated by detection of 4HPAA by GC-MS, DHPAA by LC-MS as well as production of H_2_O_2_ in a peroxidase-based fluorescent assay (Fig. 3b–d). PsTyDC6 also exhibits bifunctional AAAD activity which is indicated by the LC-MS detection of tyramine and dopamine as products of tyrosine and L-DOPA, respectively, and also by the production of downstream norlaudanosoline from L-DOPA and norcoclaurine from L-DOPA and tyrosine (Fig. 3d).

### *P. somniferum* pyruvate decarboxylase promotes an alternative 4HPAA bypass pathway

**Fig. 4:**
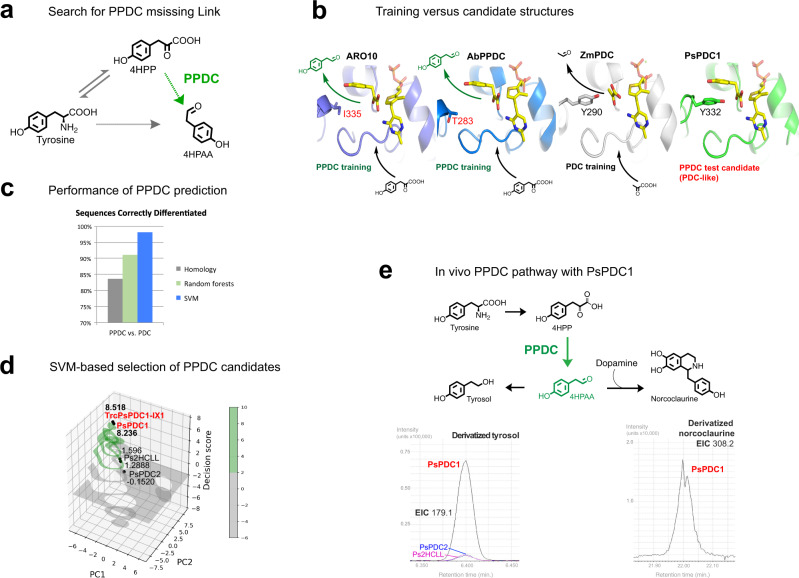
PsPDC1 promotes an alternative branch to 4HPAA for norcoclaurine production. **a** The PPDC branch pathway (green) can produce 4HPAA via 4HPP, and this missing link is also unreported in *P. somniferum*. **b** Structural comparison of classified phenylpyruvate decarboxylase (PPDC) enzymes ARO10 (purple), *Azospirillum brasilense* PPDC (AbPPDC, PDB ID: 2Q5O [https://www.rcsb.org/structure/2Q5O], blue) in comparison to typical *Zymomonas mobilis* pyruvate decarboxylase (PDC) (ZmPDC, PDB ID: 2WVA [https://www.rcsb.org/structure/2WVA], grey) and candidate PPDC sequence PsPDC1 (green). The modeled PsPDC1 active site contains Y332, which is also present in typical PDC enzymes which decarboxylate pyruvate. In this respect, the PsPDC1 active site is distinct from microbial PPDCs, which all contain smaller residues (red) in place of Y332 (*Lactococcus lactis* KdcA contains S286 corresponding to Y332). Yet, the presence of Y332 in PsPDC1 does not interfere with the docking of tyrosine into the PsPDC1 active site. **c** Cross-validation for correct assignment of PPDC model training sequences is performed using SVM models (blue), Random forests models (green), and by comparing sequence homology of each training sequence to a consensus sequence of PPDC training sequences and a consensus sequence of PDC training sequences (grey), as described in the methods section. **d** SVM-based prediction of putative PPDC sequences visualized in three dimensions by compressing high-dimensional data (Supplementary Table 3, upper table) into two dimensions (PC1 and PC2) and plotting them together with two-dimensional decision scores. Prediction spaces with two-dimensional decision scores above and below 2 are colored green and grey, respectively. Prediction score trends for truncated PsPDC1 isoform X1 (TrcPsPDC1-IX1, red), PsPDC1 (red), PsPDC2 and Ps2HCLL (2-hydroxyacyl-CoA ligase-like) are similar in high dimensional models (Supplementary Table 3). **e** PsPDC1 mediates in vivo production of 4-hydroxyphenylethanol (tyrosol) through a 4HPAA intermediate (green), in M9 medium supplemented with 1.2 mM 4HPP at 25 °C with 180 rpm shaking. Strain P1-01-AI, which contains PsPDC1 (red), mediates higher tyrosol production than that of strains P2-01-AI and P3-01-AI, which contain PsPDC2 (blue) and Ps2HCLL (magenta), respectively. PsPDC1 (red) mediates downstream production of norcoclaurine (NC) from LB supplemented with 5 mM tyrosine and 3.7 mM dopamine in strain P1-02-AI, at 20–25 °C with 180 rpm shaking. Here, tyrosol is detected after 71 h, and norcoclaurine is detected after 41 h, from filtered and dried culture medium as trimethylsilyl (TMS)-derivatives using GC-MS, as shown in the extracted ion chromatograms (EICs). Detection of PsPDC1 products is replicated in Supplementary Fig. 4.

Thiamine pyrophosphate (TPP)-dependent PPDC is an alternative to PLP-dependent AAS for the production of aryl acetaldehyde intermediates 4HPAA and DHPAA (Fig. 4). Previous reports hypothesize that *P. somniferum* should contain PPDC with specificity towards 4-hydroxyphenylpyruvate (4HPP)^21^; however, no plant protein accessions are found with the annotation of phenylpyruvate decarboxylase. In comparison to the known enzymes with PPDC activity, including *Azospirillum brasilense* ipdC^24^, *Lactococcus lactis* KdcA^25^, and yeast ARO10^26^, the active site of *P. somniferum* pyruvate decarboxylase 1 (PsPDC1) more closely resembles that of typical pyruvate decarboxylase (PDC)^27^ (Fig. 4b). Yet, in SVM prediction models built according to the methods section (Supplementary Data 3 and Supplementary Data 4), PsPDC1 appears in the PPDC prediction space (Fig. 4d and Supplementary Table 3). Two additional test candidates, *P. somniferum* PDC2 (PsPDC2) and a 2-hydroxyacyl-CoA ligase-like protein, score lower for PPDC prediction and result in lower in vivo production of downstream 4HPP decarboxylase products, in comparison to that of PsPDC1 (Fig. 4d, e, and Supplementary Fig. 4). The PPDC prediction model also suggests that truncated PsPDC1 isoform X1 (TrcPsPDC1-IX1) is a strong PPDC candidate sequence, and therefore this candidate is prepared for expression (Fig. 4d and Supplementary Table 7).

In vivo screenings with PsPDC1 reveal the alternative alkaloid route through 4HPP, and this PPDC bypass is distinct from the direct aromatic amino acid branch mediated by PsTyDC1 (Fig. 4e). Application of PsPDC1 for conversion of tyrosine through the 4HPP and 4HPAA containing pathway results in improvement in norcoclaurine titers to the >10 μM range (Fig. 4e) as estimated by GC-MS, compared the 100–200 nM range of PsTyDC1 as estimated by LC-MS (Fig. 2e and Supplementary Fig. 3).

### Automatic selection of paired CYP450 and CYP450 reductase sequences extends the 4HPAA pathway

**Fig. 5:**
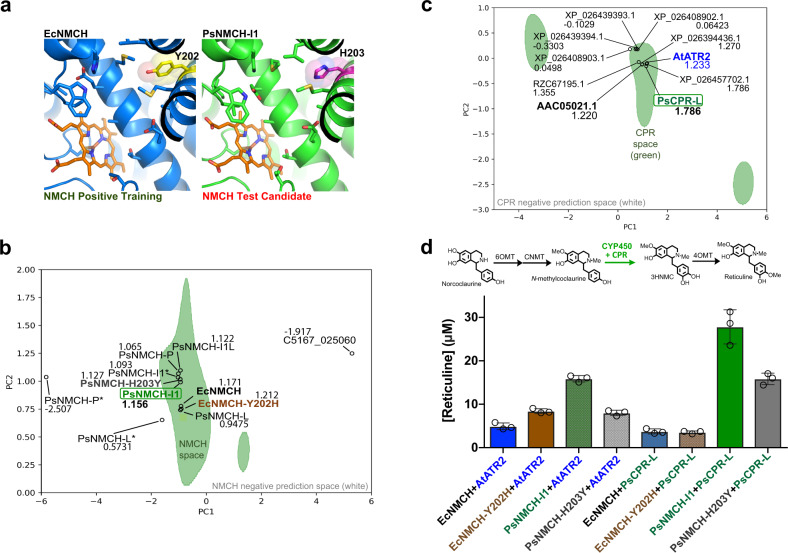
Demonstration level prediction and tuning of *P. somniferum* NMCH and CPR for improved reticuline production from norcoclaurine. **a** Comparison of active site configurations of positive training example EcNMCH (blue) with that of candidate sequence PsNMCH Isoform 1 (PsNMCH-I1, green). **b** For visual representation, two-dimensional SVM-based prediction of NMCH sequences is shown, with positive and negative prediction spaces colored green and white, respectively. Selected sequences are shown in bold. With exception to EcNMCH and EcNMCH-Y202H (brown), all points represent *P. somniferum* sequences. PCA is used to compress multi-dimensional data into two dimensions (PC1 and PC2) for the visual representation. Corresponding high-dimensional SVM results are detailed in Supplementary Table 4, and high-dimensional SVM decision scores are listed. **c** Two-dimensional SVM-based prediction of CPR sequences, with positive and negative prediction spaces colored green and white, respectively. With exception to positive training sequence AtATR2 (blue), all points represent tested *P. somniferum* sequences. PCA is used to compress multi-dimensional data into two dimensions (PC1 and PC2) for the visual representation. Corresponding high-dimensional SVM results are detailed in Supplementary Table 5, and high-dimensional SVM decision scores are listed. **d** Conversion of 1.2 mM norcoclaurine to reticuline, mediated by various combinations of NMCH and CPR, together with Ps6OMT, PsCNMT and Ps4OMT, in strains N1-01-DE3, N1-02-DE3, N1-03-DE3, N1-04-DE3, N2-01-DE3, N2-02-DE3, N2-03-DE3, N2-04-DE3 (Supplementary Table 2). Here, individual samples were analyzed 3 times (*n* = 3) to generate bar graphs in Prism 7 version 7.0d with error bars representing mean values +/− standard deviations. Source data are provided as a Source Data file.

After constructing the 4HPPA pathway to norcoclaurine, *P. somniferum* CYP450 homologs of *N*-methylcoclaurine 3-hydroxylase (NMCH) are next considered to extend this pathway from *N*-methylcoclaurine to reticuline (Fig. 5). Currently, *Saccharomyces cerevisiae* BIA productions utilize characterized *Eschscholzia californica* NMCH (EcNMCH) for conversion of *N*-methylcoclaurine to 3-hydroxy-*N*-methylcoclaurine (3HNMC)^1,16,17,28^. There are several promising *P. somniferum* CYP450 sequences annotated as NMCH based on skillful characterizations in plants^29–31^. However, the presence of many additional CYP450 homologs in the *P. somniferum* genome complicates the selection of the best candidate sequence by non-experts. To automate the selection of optimal NMCH and CPR sequences, a SVM model was trained using positive training vectors derived from plant CYP80B sequences (Supplementary Data 5). 100 *P. somniferum* CYP450 sequences were then tested against this model to assist the selection of an optimal candidate (Fig. 5b, Supplementary Table 4). As a result of this demonstration level prediction (Fig. 1c), PsNMCH Isoform 1 (PsNMCH-I1) scored high against the model and was selected.

A CYP450 reductase (CPR) redox partner for PsNMCH was selected based on the decision scores of an SVM model trained using the sequences in Supplementary Data 6. While a CPR sequence has been characterized from *P. somniferum*^32^, the referenced sequence AAC05021.1 is annotated as ‘NADPH:ferrihemoprotein oxidoreductase’, which may confuse the selection of this sequence as CPR by non-experts. Moreover, there are at least 8 other unique *P. somniferum* sequences with high CPR homology that have not been characterized. After testing the 8 additional *P. somniferum* candidates against the CPR SVM model, XP_026404029.1 is selected as a high scoring sequence (Fig. 5c and Supplementary Table 5), and observed to exhibit CPR activity (Fig. 5d). This CPR sequence is annotated as ‘NADPH-cytochrome P450 reductase-like’, and accordingly it is referred to as PsCPR-L in this manuscript.

NMCH activity is evaluated by converting norcoclaurine to stable reticuline using NMCH and CPR variants expressed together with norcoclaurine 6-*O*-methyltransferase (6OMT), coclaurine *N*-methyltransferase (CNMT) and 3-hydroxy-*N*-methylcoclaurine 4-*O*-methyltransferase (4OMT) (Fig. 5d and Supplementary Table 2). *N*-methylcoclaurine accumulates much more than other intermediates in this system, and therefore stable reticuline titers should reflect the activity of the NMCH bottleneck. In this system, PsNMCH-I1 affords higher amounts of reticuline than that of EcNMCH, when paired with either PsCPR-L or AtATR2 (Fig. 5d). PsNMCH-I1 pairs best with PsCPR-L from the same species, resulting in the highest amount of reticuline. On the other hand, reticuline production with EcNMCH is best with AtATR2 pairing, with no improvement from PsCPR-L pairing.

Just one residue difference is observed when comparing the binding pockets of PsNMCH and EcNMCH: PsNMCH-H203 versus EcNMCH-Y202 (Fig. 5a). SVM prediction of PsNMCH-H203Y and EcNMCH-Y202H sequences results in lower and higher decision scores in comparison to those of wild-type sequences, respectively (Fig. 5b and Supplementary Table 4), indicating that the SVM model is able to identify this key residue as an important feature. Consistent with this prediction, transplantation of EcNMCH-Y202 into engineered PsNMCH-H203Y results in lower reticuline, and transplantation of PsNMCH-H203 into engineered EcNMCH-Y202H results in higher conversion of norcoclaurine to reticuline when paired with AtATR2.

Early in vivo tests of PsNMCH-I1 without a CPR redox partner in *E. coli* did not result in detectable NMCH activity, but L-DOPA production from tyrosine was detected (Supplementary Fig. 3). This led us to hypothesize that PsNMCH-I1 might also have potential tyrosine 3-monooxygenase activity; however, the observed L-DOPA production is probably more likely to be mediated by native *E. coli* HpaBC. To further clarify this important missing link in *P. somniferum*, the candidate CYP450 monooxygenase sequences are also explored as potential tyrosine 3-monooxygenase templates (Supplementary Table 6). Here, the candidate sequences are tested against an SVM model trained using plant CYP76AD sequences as positive examples (Supplementary Data 7), and a combined SVM model trained with plant CYP76AD, CYP98A3 and CYP199A2 sequences as positive examples (Supplementary Data 8)^33–35^. CYP98A2-like (XP_026403623.1), geraniol 8-hydroxylase-like (XP_026409442.1) and flavonoid 3,5-hydroxylase 1-like (XP_026378021.1) sequences appear as prime targets with relatively high scores in the positive prediction space of both high-dimensional models of Supplementary Table 6.

### Emergence of dual norcoclaurine and norlaudanosoline pathways via expression of *P. somniferum* decarboxylases

**Fig. 6:**
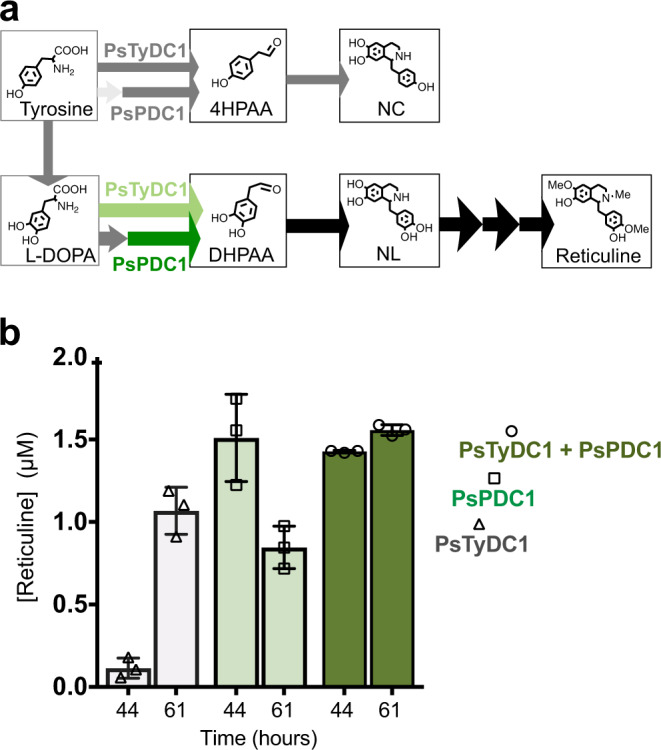
PsPDC1 and PsTyDC1 promote the norlaudanosoline pathway from L-DOPA to reticuline. **a** Pathway expansion of the *P. somniferum* 4HPAA pathway to a dual norcoclaurine (NC) and norlaudanosoline (NL) pathway. **b** Strains T1-10-DE3, P1-02-AI and P1-04-AI contain PpDDC, PsONCS3, Cj6OMT, CjCNMT, and Cj4OMT in addition to PsTyDC1 (grey), PsPDC1 (light green) and PsTyDC1 + PsPDC1 (dark green), respectively. Cultures were grown to high density in TB before addition of inducing agent, L-DOPA and ascorbate according to the methods section. 61 h after addition of L-DOPA substrate, PsPDC1-mediated reticuline titers declined, likely due to oxidative degradation. Replicate samples of filtered culture medium were analyzed with CE-MS (*n* = 3). Here, 3 samples from individual cultures (*n* = 3) were analyzed to generate bar graphs in Prism 7 with error bars representing mean values +/− standard deviations. Source data are provided as a Source Data file.

PsTyDC6 shares over 98% sequence identity with PsTyDC1 and is able to convert tyrosine and L-DOPA to norcoclaurine and norlaudanosoline (Fig. 3). Furthermore, co-expression of PsTyDC1 with TfNCS, PsNMCH-I1, Cj6OMT, CjCNMT, and Cj4OMT, results in a plant-gene only dual pathway through 4HPAA and DHPAA to norcoclaurine and reticuline (Supplementary Fig. 3). Therefore, the potential DHPAAS activity of PsTyDC1 is further explored to construct combined norcoclaurine and norlaudanosoline pathways (Fig. 6a). At the same time, PsPDC1 is also explored as a mediator of DHPAA production via decarboxylation of transaminated L-DOPA.

After incorporating L-DOPA decarboxylase (DDC) from *Pseudomonas putida* (PpDDC) for in vivo dopamine production and optimization in Terrific Broth (TB), PsPDC1 and PsTyDC1 containing strains produce reticuline from L-DOPA via the DHPAA pathway, with titers reaching the μM range (Fig. 6b). Previously, a single strain containing DHPAAS, 6OMT, CNMT, and 4OMT only produced reticuline titers of 0.2 μM from L-DOPA^7^. These results suggests that PsPDC1 can produce DHPAA from 3,4-dihydroxyphenylpyruvic acid (DHPP) that is supplied by L-DOPA transamination, and that PsPDC1 works synergistically with PsTyDC1 at later production times to promote high reticuline production in *E. coli*. Accordingly, combinations of PPDC and AAS are next explored to improve BIA titers.

### Expanding the prediction models towards template enzyme engineering

**Fig. 7:**
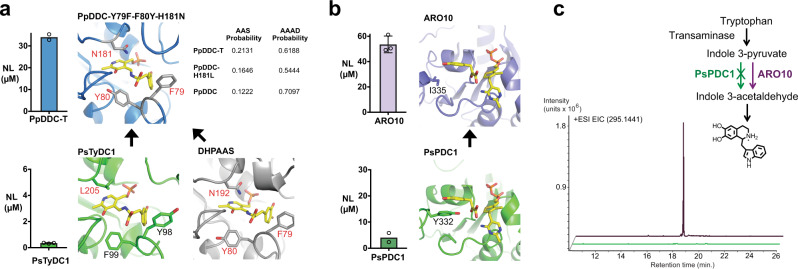
Replicating norlaudanosoline pathways using homologous enzyme templates. **a** PsTyDC1 (green) is exchanged with an engineered PpDDC template (blue) via three active site gain-of-function substitutions, Y79F, F80Y, and H181N, to promote DHPAAS activity. In accordance with the resulting increase in AAS activity, these three substitutions result in increased SVM probability scores for AAS prediction, and reduced SVM probability scores for AAAD prediction. Norlaudanosoline (NL) production from L-DOPA was compared using PsTyDC1 in strain T1-10-DE3 (*t* = 44 h, *n* = 3) and PpDDC-Y79F-F80Y-H181N (PpDDC-T) in strain DT-02-DE3 (*t* = 40.5 h, *n* = 2). Culture conditions for each strain are described in the methods section. **b** PsPDC1 (green) is exchanged with *S. cerevisiae* ARO10 (purple) for higher PPDC activity in *E. coli*. Production of norlaudanosoline (NL) from L-DOPA by PsPDC1 in strain P1-02-AI is shown (*t* = 44 h, *n* = 2). Production of norlaudanosoline (NL) from L-DOPA and dopamine by ARO10 in strain A1-01-DE3 is compared (*t* = 44 h, *n* = 3). Culture conditions are described in the methods section. For panels **a** and **b**, Samples from individual cultures were analyzed two or three times (*n* = 2 or *n* = 3) to generate bar graphs in Prism 7, with error bars representing mean values +/− standard deviations. **c** Strain A1-01-DE3 containing ARO10 (purple) metabolizes tryptophan in TB medium to produce an indole 3-acetaldehyde derived indole alkaloid byproduct (*t* = 61 h), as indicated by the extracted ion chromatogram (EIC). Strain P1-02-AI containing PsPDC1 (green) did not readily convert indole 3-pyruvate to indole 3-acetaldehyde, as indicated by no detectable indole alkaloid byproduct (*t* = 61 h). Source data are provided as a Source Data file.

The characterizations of PsTyDC1, PsTyDC6, and PsPDC1 indicate that these enzymes promote dual pathways in *E. coli*. However, the activity of PsTyDC1 is low under the conditions tested, while preliminary experiments show that PsPDC1 and TrcPsPDC1-IX1 expression in *E. coli* is unstable and toxic. Therefore, in order to quickly achieve in vivo titers high enough for dynamic metabolomic profiling, dual norcoclaurine and norlaudanosoline pathways are re-explored using homologous enzyme templates with stable expression in *E. coli* (Fig. 7). The concept of template enzyme engineering refers to the approach where useful features are identified from a specialized enzyme and those features are transplanted into a related template to confer some advantages. This is illustrated with the above EcNMCH-Y202H substitution where the corresponding H203 residue from PsNMCH is substituted to improve EcNMCH as the template enzyme. To further develop this methodology, the SVM enzyme selection algorithm is applied to evaluate multiple enzyme engineering substitutions for highly active template sequences, using PpDDC as a specific example (Fig. 7a).

**Fig. 8:**
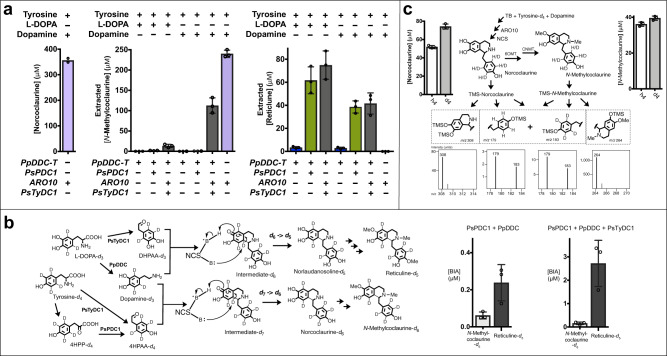
Optimization of norcocluarine, reticuline, and *N*-methylcoclaurine production for analysis of flux through hybrid pathways. **a** PpDDC-Y79F-F80Y-H181N (PpDDC-T) and PsPDC1 containing strain P1-07-AI (olive green) prefers the norlaudanosoline containing pathway. Combination of PpDDC-T, ARO10 and PsTyDC1 in strain A1-06-AI (dark grey) promotes both norcoclaurine and norlaudanosoline containing pathways. ARO10 expressing strain A1-01-DE3 (light purple) converts tyrosine and dopamine to norcoclaurine and *N*-methylcoclaurine. *N*-Methylcoclaurine and reticuline were extracted with ethyl acetate from cultures 40 h after addition of tyrosine together with L-DOPA or dopamine. Tested strains P1-06-DE3 (blue), P1-07-AI (olive green), A1-06-AI (dark grey) and A1-01-DE3 (light purple) each contain Cj6OMT, CjCNMT, Cj4OMT, NCS, plus the indicated genes of the bottom 4 rows. P1-06-DE3 and P1-07-AI contain the same genes, but P1-06-DE3 was induced with only IPTG, without including arabinose for PsPDC1 expression. Cultures containing PpDDC-T and L-DOPA were supplemented with additional sodium ascorbate. The BL21(AI) derived strain P1-07-AI was induced with IPTG and arabinose. For improved *N*-methylcoclaurine production, A1-01-DE3 was supplemented with the aldehyde reductase/dehydrogenase inhibitor gossypol. Additional culture conditions are described in the methods section. Extracted *N*-methylcoclaurine and reticuline were TMS-derivatized and analyzed with GC-MS (*t* = 40 h, *n* = 3). After extraction, cultures were stored at 4 °C and stable norcoclaurine titers from culture medium were analyzed with LC-MS (*n* = 2). **b** Isotope profiling of strains P1-02-AI (expressing PsPDC1) and P1-04-AI (expressing PsPDC1 and PsTyDC1), which produce *N*-methylcoclaurine-*d*_6_ (P1-02-AI - 62 nM, P1-04-AI 160 nM) and reticuline-*d*_5_ from tyrosine-*d*_4_ and L-DOPA-*d*_3_ (*t* = 61 h, *n* = 3). There is a synergistic improvement in BIA production when combining PsPDC1 and PsTyDC1. Here, NCS catalyzes the loss of a deuterium from in vivo generated dopamine-*d*_*3*_. **c** For tracing aromatic isotope flux from tyrosine to norcoclaurine and *N*-methylcoclaurine, alkaloids were extracted with ethyl acetate from the A1-01-DE3 culture 40 h after addition of tyrosine-*d*_*3*_ and dopamine, according to the methods section. Extracted alkaloids were TMS-derivatized and analyzed with GC-MS (*n* = 3). After extraction, cultures were stored at 4 °C and stable BIA titers from culture medium were analyzed with CE-MS (*n* = 3); the fraction of labeled BIA-*d*_4_ and unlabeled BIA from natural tyrosine in the rich TB broth can be quantified. With exception to unlabeled norcoclaurine (*n* = 2), all other individual samples were analyzed 3 times (*n* = 3) to generate bar graphs in Prism 7, with error bars representing mean values +/− standard deviations. Source data are provided as a Source Data file.

AAS activity analogous to that of PsTyDC1 could be engineered into the bacterial PpDDC template by transplanting DHPAAS specific catalytic residues F79, Y80 and N181 (PpDDC numbering). Rationally engineered PpDDC-Y79F-F80Y-H181N mediates improved norlaudanosoline production in *E. coli* (Fig. 7a). Switching from PsPDC1 to a *S. cerevisiae* ARO10 template is observed to improve in vivo turnover of both DHPP (Fig. 7b) and 4HPP (Fig. 8a), in comparison to corresponding strains containing PsPDC1. However, the high activity of ARO10 may come at a specificity tradeoff, as the production of additional aromatic keto acid-derived alkaloids result from ARO10 expression (Fig. 7c).

Combinations of natural and analogous enzyme templates result in improved *E. coli* BIA production (Fig. 8a and Supplementary Table 2). Expression of PpDDC-Y79F-F80Y-H181N together with PsPDC1 in strain P1-07-AI selectively promotes the DHPAA pathway in the presence of tyrosine and L-DOPA to produce 61.8 µM reticuline, while the application of ARO10 in strain A1-01-DE3 selectively favors the 4HPAA pathway in the presence of tyrosine and dopamine to produce 356 µM norcoclaurine and 240 µM *N*-methylcoclaurine. Dual pathway production of 112 µM *N*-methylcoclaurine and 74.9 µM reticuline is promoted through the combination of PpDDC-Y79F-F80Y-H181N, ARO10 and PsTyDC1 in strain A1-06-AI.

### Dynamic metabolomic profiling of AAS and PPDC branch pathways

By tracing the turnover of isotope-labeled precursors and measuring the resulting fractions of isotope-labeled intermediates, metabolic flux can be observed, and this approach is referred to as dynamic metabolic profiling^5,36,37^. While multiple reaction monitoring (MRM) with LC-MS is sensitive, this method does not readily detect isotope-labeled intermediates. After improving BIA titers to μM levels suitable for quantification with high-resolution CE-MS, isotope tracing experiments could be performed. Combinations of PsPDC1, ARO10, PsTyDC1 and PpDDC produce various labeling patterns: tyrosine-^13^*C* to BIA-^13^*C*_2,_ L-DOPA-*d*_3_ with tyrosine-*d*_4_ to *d*_6_-labeled BIA, L-DOPA-*d*_3_ to *d*_5_-labeled BIA, L-DOPA-*d*_3_ with dopamine-*d*_2_ to *d*_5_-labeled BIA, tyrosine-*d*_4_ with dopamine-*d*_2_ to *d*_6_-labeled BIA, and tyrosine-*d*_4_ with dopamine to *d*_4_-labeled BIA (Fig. 8b, c, and Supplementary Fig. 5). The loss of a ring deuterium atom during NCS-mediated condensation of aryl acetaldehydes with ring-labeled dopamine is consistent with the reported NCS mechanism (Fig. 8b and Supplementary Fig. 5d)^38,39^; this kind of mechanism-directed deuterium labeling pattern has not been reported for the tracing of BIA^40–42^. Isotope tracing from L-DOPA-*d*_3_ to *d*_5_-labeled BIA supports the bifunctional decarboxylase and oxidative deamination activities of PpDDC-Y79F-F80Y-H181N (Supplementary Fig. 5d). Improvement of *N*-methylcoclaurine-*d*_6_ and reticuline-*d*_5_ production via PsTyDC1 in addition to PsPDC1 again demonstrates the synergistic combination of these distinct enzymes (Fig. 8b). Moreover, amounts of *N*-methylcoclaurine-*d*_6_ and reticuline-*d*_5_ relative to their respective precursors norcoclaurine-*d*_6_ and norlaudanosoline-*d*_5_ (Supplementary Fig. 5b, c) show the bottleneck of the *S*-adenosylmethionine (SAM)-dependent methylation of deuterium-labeled BIA. Furthermore, isotope tracing from tyrosine-^13^*C* supports that PsPDC1 and ARO10 are converting isotope-labeled 4-hydroxyphenylpyruvic acid (4HPP) to downstream BIA (Supplementary Fig. 5a).

Dynamic metabolomic profiling of mixed fractions of unlabeled and labeled BIA, could be performed with high-titer norcoclaurine-*d*_4_ and *N*-methylcoclaurine-*d*_4_ production (Fig. 8c). In this case, a higher fraction of *d*_4_-labeled norcoclaurine relative to *d*_4_-labeled *N*-methylcoclaurine is consistent with the SAM-dependent methyltransferase bottleneck observed previously^1,7^.

## Discussion

This report demonstrates that machine learning can uncover missing link enzymes with direct applications to biomanufacturing. While previous studies have also reported machine learning for enzyme prediction, these examples were never applied to the discovery of uncharacterized enzymes^43–47^. In the report by Li et al., prediction of active glutaminase and aurora kinases B were used as examples to verify their algorithm, however, this test data was obtained from the previous publications^45^. On the other hand, a study by Tietz et al. applied SVM to predict ribosomally synthesized and post-translationally modified peptides precursor peptides, of which some were validated experimentally^48^. Similarly, the current study demonstrates the paired prediction and experimental investigation of four kinds of plant enzymes. Furthermore, the possibility to engineer artificial enzymes is demonstrated by prediction of PsNMCH-H203Y, EcNMCH-Y202H (Fig. 5) and PpDDC-Y79F-F80Y-H181N (Fig. 7a), where scores are in agreement with in vivo test results. Therefore, the SVM prediction models of this study (Supplementary Tables 1 and 3–6) can readily enable the discovery and engineering of specialized carboxy-lyases (EC 4.1.1.X), CYP450s (EC 1.14.X.X) and CPRs (EC 1.6.2.4). While the current machine learning method is shown to be superior to homology-based selection of PLP-dependent AAS and TPP-dependent PPDC sequences (Fig. 2c and Fig. 4c), additional studies should be pursued to demonstrate improved selection for other classes of enzymes.

PsPDC1 shows potential for in vivo PPDC activity and contains active site residue Y332, which is also present in ZmPDC that is known to only convert small non-aromatic substrates. This active site tyrosine is substituted with smaller residues in characterized yeast and bacterial PPDC enzymes (Fig. 4b), and therefore the structural basis of plant PPDC substrate recognition appears to be determined by other factors. Species-by-species variation in functional residues is also seen with the evolution of AAS variants throughout insects and plants. Insects have evolved a histidine to asparagine active site switch, corresponding to residue 192 of DHPAAS, to promote AAS activity essential for their survival (Fig. 2b)^7,12^. In the plant homologs, tyrosine is commonly substituted with a more hydrophobic phenylalanine (residue 346 of *Petroselinum crispum* 4HPAAS, Fig. 2b) to switch from AAAD to AAS activity. Yet the active site of PsTyDC6 resembles that of typical AAAD while still promoting AAS activity. These results with PsPDC1 and PsTyDC6 indicate that specialized PPDC and AAS activities may exist in other plant sequences that resemble typical carboxy-lyases. This insight also suggests that combinations of subtle structural features or emergent properties may be underlying the specialized activities of select plant carboxy-lyases. Accordingly, machine learning offers advantages over structural analysis to identify elusive emergent features in enzymes with specialized functions that cannot be predicted from structure or homology alone.

Transplantation of discovered functional residues into high-activity microbial templates is an effective strategy for improving bioproduction, as demonstrated by PpDDC-Y79F-F80Y-H181N with transplanted DHPAAS active site residues. In this example, the design of three amino acid substitutions, including the most critical H181 substitution that corresponds to PsTyDC1-L205, could be guided with the SVM prediction algorithm. Improved protein stability, removal of regulation/inhibition, and improved expression in host cells are additional factors that might contribute to improved templates. While PpDDC-Y79F-F80Y-H181N favors the DHPAA pathway, PsTyDC6 is capable of mediating both DHPAA and 4HPAA containing pathways. Similarly, expression of PsPDC1 and ARO10 are observed to promote the conversion of 4HPP to 4HPAA and DHPP to DHPAA, but strains expressing PsPDC1 favor the DHPAA containing pathway under the conditions tested (Fig. 8a, b). This illustrates that the control of flux through the norcoclaurine route versus the norlaudanosoline route may be controlled by the selection of specific PPDC and AAS templates.

Many sophisticated studies have elucidated key steps of plant BIA metabolism, and as a result many of the discovered enzymes have enabled the production of key intermediates and downstream medicinal compounds in yeast and *E. coli*^21,28–32,49–52^. For high-level production of the key intermediate reticuline, current studies require NCS and plant OMTs, but their selection has generally been limited to a few sequences from *P. somniferum, C. japonica* and *T. flavum*^1,7,13–17^. Yeast studies have focused on the natural norcoclaurine route, and require the CYP450 enzyme NMCH to complete the pathway to reticuline. Yet in the recent *S. cerevisiae* reports, the first well-characterized NMCH from *E. californica* continues to be selected^1,16,17,28^. In light of this, machine learning-based enzyme prediction offers great potential to expand the choice of additional homologous sequences that might further boost product titers. For example, the predicted pathways with PsNMCH-I1 paired with PsCPR-L and EcNMCH-Y202H paired with AtATR2 produce higher reticuline titers than that of the conventional EcNMCH and AtATR2 combination.

For production in *E. coli*, Matsumura et al. were able to achieve 160 mg/L (*S*)-reticuline using a MAO-dependent and NMCH-independent pathway through norlaudanosoline in *E. coli*^13^. In contrast, establishing the natural norcoclaurine pathway in *E. coli* produces high levels of extracted norcoclaurine (96.7 mg/L) and *N*-methylcoclaurine (71.8 mg/L) (Fig. 8a). Although yeast is better suited than *E. coli* for the expression of CYP450s including NMCH, production of expensive *N*-methylcoclaurine does not require NMCH. In addition, the dual pathway strain of the current study produces 24.6 mg/L reticuline and 33.6 mg/L *N*-methylcoclaurine (Fig. 8a).

Considerable experimental evidence has indicated that plant BIA pathways proceed through norcoclaurine and that norlaudanosoline is not a natural metabolite of *P. somniferum*^50,52^. In contrast, the current characterizations of PsTyDC1, PsTyDC6 and PsPDC1 show that these enzymes promote the production of both norlaudanosoline and norcoclaurine in *E. coli*. This suggests that the norlaudanosoline pathway might also occur in plants; however, the precise effects of PsTyDC1^53^, PsTyDC6^53^ and PsPDC1 within the natural pathway should be carefully elucidated in plants before drawing any final conclusions.

A dual pathway to norcoclaurine and norlaudanosoline in *E. coli* offers advantages for utilization of tyrosine, and for improving amounts of unstable aryl acetaldehydes relative to dopamine. Accordingly, increased aryl acetaldehyde production by synergistic expression of PsPDC1 together with PsTyDC1 results in increased reticuline through an enhanced norlaudanosoline pathway (Fig. 6 and Fig. 8b). These pathways were further characterized by dynamic metabolomic profiling, an approach that can readily identify bottleneck targets for increasing metabolic flux to target compounds. In conclusion, machine learning discovery of missing links and homologous enzyme templates is now a realistic approach for assembling alternative routes and relieving bottlenecks in improved metabolic pathways.

## Methods

### Machine learning prediction

Support vector machine (SVM) Enzyme-models were built from enzyme amino acid sequence information using scikit-learn version 0.21.3^8^, and the resulting computer code was made publically available [https://github.com/nwatanbe/SVM_E_model]. Aromatic amino acid decarboxylase (AAAD), aromatic acetaldehyde synthase (AAS, previously referred to as aromatic aldehyde synthase) and phenylpyruvate decarboxylase (PPDC) prediction models were trained with vectors generated by PROFEAT^54^. AAAD positive training sequences include L-DOPA decarboxylase (DDC) and other typical PLP-dependent carboxy-lyases that decarboxylate aromatic amino acids (Supplementary Data 1). The AAAD positive examples all contain a catalytic histidine, corresponding to H181 of PpDDC (Fig. 2b). Characterized PsTyDC9^22^ is included as a positive AAAD training sequence to ensure there is no bias towards AAS prediction. For AAS models, the positive training examples consist of sequences with homology to known plant-type and insect-type AAS enzymes, including *Petroselinum crispum* 4HPAAS (Pc4HPAAS) and insect DHPAAS (Supplementary Data 2). Insect-type AAS sequences are classified based on the presence of N192 (insect DHPAAS numbering), and plant-type AAS enzymes are classified based on the presence of F346 or V346 (Pc4HPAAS numbering).

For PPDC prediction models, positive training vectors included sequences annotated as PPDC and indolepyruvate decarboxylase (Supplementary Data 3 and Supplementary Data 4). Since all current database sequences annotated as phenylpyruvate decarboxylase are from bacteria and fungi (plus 1 from Archaea), typical pyruvate decarboxylase (PDC) sequences also had to be included in the first prediction model (Supplementary Table 3, upper table). After discovering PsPDC1, a rose PPDC sequence was found from continuous literature searches, although its protein accession (BAU70033.1 [https://www.ncbi.nlm.nih.gov/protein/BAU70033.1]) is annotated as ‘pyruvate decarboxylase’^55^. A second PPDC specific SVM model was therefore built by training with 19 homologous plant sequences in the same phylogenetic clade as rose PPDC as positive training sequences and 3 negative training sequences which were curated as plant PDC, as suggested by the results of a previous report^56^.

Positive training sequences from AAS and PPDC models were included as negative training sequences for the AAAD model; positive training sequences from AAAD and PPDC models were included as negative training sequences for the AAS model; and positive training sequences from AAS and AAAD models were included as negative training sequences for PPDC models. For all models, general negative training sequences included *E. coli*, *S. cerevisiae* and *A. thaliana* enzymes, excluding sequences classified in the positive training group.

Cytochrome P450 (CYP450) prediction models were trained with vectors generated by ProtVec^57^. To clarify potential *N*-methylcoclaurine 3-hydroxylase (NMCH) activities of CYP450 monooxygenases, SVM models were trained with CYP80B sequences as positive examples (Supplementary Data 5). CYP450 reductase (CPR) prediction models were trained using sequences listed in Supplementary Data 6. To clarify potential tyrosine 3-monooxygenase activities, SVM models were trained with sequences related to CYP76AD, CYP98A3 and CYP199A2 as positive examples (Supplementary Data 7 and Supplementary Data 8). CYP76AD, CYP98A3 and CYP199A2 enzymes are reported to mediate aromatic hydroxylation of tyrosine and the similarly sized substrate coumaric acid^33–35^.

Prediction models were first built with high-dimensional vectors. Cross validation of all high-dimensional SVM models resulted in F-scores above 0.96. Candidate sequences were selected based on high-dimensional scores. Two-dimensional and three-dimensional plots were used for visual representation of data in Figures. For two-dimensional plots, high-dimensional vectors were compressed to 2 dimensions using principal component analysis (PCA). 2-dimensional SVM models were then built derived from the PCA compressed vectors. SVM and PCA from the scikit-learn library were used^58^. The three-dimensional SVM plot in Fig. 4d was adopted from an SVM illustration by Dr. Saptashwa Bhattacharyya [https://towardsdatascience.com/visualizing-support-vector-machine-decision-boundary-69e7591dacea]. Compressed two-dimensional decision scores from the combined model (Supplementary Table 3, upper table) are used as the third dimension of Fig. 4d.

Random forests E-models were built from enzyme amino acid sequence information using scikit-learn version 0.21.3^8^, with the same datasets and feature extractions as that of the corresponding SVM models. As an additional benchmark, machine learning differentiation of AAS versus AAAD sequences, and PPDC versus PDC sequences, was compared to differentiation based on homology to consensus sequences. To do this, consensus sequences were generated for each group of training sequences (AAS, AAAD, PPDC and PDC), by selecting the amino acid of maximum frequency at each position. If a training sequence has higher sequence identity to the consensus sequence of its correct group, compared to that of its related group, then it was counted as a correct prediction by homology.

Training sequences, cross-validation F-scores and additional parameters for high-dimensional models are available in the Supplementary Data files.

### Protein structural modeling and docking analysis

Homology models were built with Modeller^59^ run in UCSF Chimera (candidate version 1.15), using template structures of highly similar proteins from the Protein Data Bank [https://www.rcsb.org]^7^. Multimeric structures and ligands were first prepared in PyMOL version 1.8.7.0. Structures were refined and prepared for docking analysis using Molecular Operating Environment (MOE) version 2020.0901^7^.

### Materials and reagents

KOD -Plus- and Ex-Taq HS DNA polymerases were purchased from Toyobo (Tokyo, Japan) and Takara (Tokyo, Japan), respectively. A-attachment Mix was purchased from Toyobo. Primers were ordered from Eurofins Genomics (Tokyo, Japan). A DNA ligation kit and JM109 chemical competent cells were purchased from Takara. The QIAprep Spin Miniprep Kit was obtained from Qiagen (Hilden, DE). BL21(DE3) and BL21-AI competent cells were purchased from Thermo Fisher Scientific (Waltham, MA, USA). Rosetta gami 2 cells were purchased from Sigma-Aldrich (St. Louis, MO, USA). All restriction endonucleases were purchased from New England Biolabs (NEB, Ipswich, MA, USA). Antibiotics were purchased from Nacalai Tesque (Kyoto, Japan), Sigma-Aldrich and FUJIFILM Wako Pure Chemical (Osaka, Japan). Growth medium components were purchased from BD (Franklin Lakes, NJ, USA) and Nacalai Tesque. The IMPACT system, with pTXB1 and pTYB21 vectors, and chitin resin, was obtained from NEB. Amicon Ultra centrifugal filters were obtained from Merck-Millipore (Darmstadt, Germany). The Fluorimetric Hydrogen Peroxide Assay Kit was from Sigma-Aldrich. Amplex Red (10-acetyl-3,7-dihydroxyphenoxazine) peroxidase substrate was from Thermo Fisher (Waltham, MA, USA). L-DOPA and dopamine were purchased from Tokyo Chemical Industry (TCI, Tokyo, Japan). 4-hydroxyphenylpyruvic acid was from Sigma-Aldrich. L-Tyrosine and L-ascorbic acid sodium salt were obtained from Nacalai Tesque. Analytical standards and isotopes were purchased from Santa Cruz Biotechnology (Dallas, TX, USA), Toronto Research Chemicals (New York, ON, Canada), ALB Technology (Kuala Lumpur, Malaysia), Sigma-Aldrich and Cambridge Isotope Laboratories (Tewksbury, MA, USA). *N*,*O*-Bis(trimethylsilyl)trifluoroacetamide (BSTFA) and *N*-methyl-*N*-(trimethylsilyl)trifluoroacetamide (MSTFA) were obtained from GL Sciences (Tokyo, Japan). 1,4-dithiothreitol (DTT) and pyridine were obtained from FUJIFILM Wako Pure Chemical. Chlorotrimethylsilane (TMS-Cl) was from Alfa Aesar (Haverhill, MA, USA) and methoxyamine hydrochloride was from MP Biomedicals (Irvine, CA, USA).

### Preparation of plasmids

Constructed plasmids (Supplementary Table 7) were transformed into JM109 chemically competent *E. coli* (Takara). Transformants were grown on LB-agar plates supplemented with the appropriate antibiotics at 30–37 °C. Positive clones were screened using colony PCR and target plasmids were purified using a QIAprep Miniprep Kit (Qiagen). Plasmids were then sequenced using primers listed in Supplementary Table 8, by Eurofins Genomics, or by using a BigDye Terminator v3.1 cycle-sequencing kit and a 3500xL Genetic Analyzer from Applied Biosystems (Foster City, CA, USA).

### Preparation of predicted candidate genes

Full-length *P. somniferum PsTyDC1* native coding sequence was synthesized by Integrated DNA Technologies (IDT, Coralville, IA, USA). Codon optimization of *PsONCS3* and *TfNCS* nucleotide sequences^60^ for expression in *E. coli* was assisted by Codon Optimization OnLine (COOL)^61^, resulting in the coding sequences shown in Supplementary Table 9, and the selected sequences were synthesized by IDT. The native sequence of full-length *P. somniferum NMCH isoform 1 (PsNMCH-I1)* was also synthesized by IDT.

Native coding sequences of full-length *PsPDC1*, full-length *Ps2HCLL*, and N-terminal truncated *PsPDC2* were synthesized and cloned into pBAD-DEST49 (LifeSensors Inc., Malvern, PA, USA) via the Gateway cloning system by GeneArt (Invitrogen, Waltham, Massachusetts, USA). Native coding sequences of full-length *EcNMCH, AtATR2*, and *P. somniferum CPR-like (PsCPR-L)* were synthesized and subcloned into the pMA vector by GeneArt (Invitrogen). Native coding sequences of full-length *PsTyDC6* and N-terminal truncated *PsPDC1-IX1* were synthesized and cloned into pTYB21 (NEB) by GenScript (Piscataway, NJ, USA).

### Construction of pACYC-3CjMTs-DDC vectors

The pACYC184-derived vectors containing *Coptis japonica 4OMT*, *CNMT*, *6OMT* (pACYC184-Cj4OMT-CjCNMT-Cj6OMT), and *PpDDC* (pACYC184-Cj4OMT-CjCNMT-PpDDC-Cj6OMT) were obtained from the laboratory of Professor Hiromichi Minami at Ishikawa Prefectural University^13,14^. Active site mutations were introduced into *PpDDC* in pACYC184, by way of site-directed mutagenesis using PCR with primers shown in Supplementary Table 8.

### Construction of subcloning vectors and mutations

To construct subcloning vectors for synthetic genes (*PsONCS3*, *TfNCS*, *PsTyDC1*, *CjNCS*, *PsNMCH-I1*, *EcNMCH, PsCPR-L*, *AtATR2*), and PCR amplified *PpDDC* and *ARO10* (amplified from pGK424-ARO10^62^), 3′ end A-protrusions were added to each DNA fragment using A-attachment Mix (Toyobo).

Gene mutations were generated using site-directed mutagenesis by PCR with primers listed in Supplementary Table 8. *PsTyDC1* mutations (L205H and Y98F-F99Y-L205N) were generated in subcloning vectors by PCR. *PpDDC* mutations (H181L, H181L-G344S, Y79F-F80Y-H181N, Y79F-F80Y-H181N-G344S) were generated by PCR. The *EcNMCH* mutation (Y202H) and *PsNMCH-I1* mutation (H203Y) were generated in subcloning vectors by PCR. pBad-PsPDC1-His, pBad-PsPDC2-His and pBad-Ps2HCLL-His were generated by removal of a stop codon with PCR.

### Construction of alkaloid production vectors

A *PsONCS3*^60^ containing DNA fragment was obtained from NcoI and BamHI digestion of the *PsONCS3* subcloning vector, and then cloned into pCDFDuet-1 via the NcoI and BamHI restriction sites to produce pCDFD-PsONCS3. A *TfNCS* containing DNA fragment was obtained from NcoI and BamHI digestion of the TfNCS subcloning vector, and then cloned into pCDFDuet-1 via the NcoI and BamHI restriction sites to produce pCDFD-TfNCS.

DNA fragments of *PsTyDC1* were obtained from NdeI and XhoI digestion of *PsTyDC1* subcloning vectors, and then cloned into pCDFDuet-1-PsONCS3 via NdeI and XhoI restriction sites to produce pCDFD-PsONCS3-PsTyDC1. The *PsTyDC1* containing gene fragments were also cloned into pCDFDuet-1-TfNCS via NdeI and XhoI sites to produce pCDFD-TfNCS-PsTyDC1. Digestion of the *PsTyDC1* subcloning vector with NdeI and SapI was used to clone into pTXB1 via NdeI and SapI, resulting in pTXB1-PsTyDC1. To produce pTYB21-PsTyDC1, pTYB21-PsPDC1, pTYB21-PsPDC2 and pTYB21-Ps2HCLL, *PsTyDC1, PsPDC1*, *Ps2HCLL*, and N-terminal truncated *PsPDC2* were PCR amplified and cloned into pTYB21 digested with SapI and BamHI via Gibson assembly (NEB)^63^.

*EcNMCH* and *EcNMCH-Y202H* gene fragments were digested with SalI and NotI in subcloning vectors and then cloned into pCOLADuet-1 via the SalI and NotI restriction sites. *AtATR2 and PsCPR-L* fragments were next digested from the subcloning vectors using NdeI and XhoI, and then cloned into pCOLAD-EcNMCH and pCOLAD-EcNMCH-Y202H via the NdeI and XhoI restriction sites to produce pCOLAD-EcNMCH-AtATR2, pCOLAD-EcNMCH-Y202H-AtATR2, pCOLAD-EcNMCH-PsCPR-L and pCOLAD-EcNMCH-Y202H-PsCPR-L.

The DNA fragment encoding *PsNMCH-I1* with a truncated N-terminal, was digested by NotI and XhoI from the subcloning vector and then cloned into a pACYC184 derived vector containing *C. japonica 4OMT*, *CNMT*, and *6OMT* via Not I and Xho I restriction sites to produce pACYC-3CjMTs-PsNMCH. Truncated *PsNMCH-I1* and truncated *PsNMCH-H203Y* gene fragments were PCR amplified from subcloning vectors and then cloned into pCOLAD-EcNMCH-PsCPR-L digested with BamHI and NotI via Gibson assembly to produce pCOLAD-PsNMCH-PsCPR-L and pCOLAD-PsNMCH-H203Y-PsCPR-L. Truncated *PsNMCH-I1* and truncated *PsNMCH-H203Y* gene fragments were also digested with NcoI and NotI and cloned into pCOLAD-EcNMCH-AtATR2 digested with NcoI and NotI to produce pCOLAD-PsNMCH-AtATR2 and pCOLAD-PsNMCH-H203Y-AtATR2.

DNA fragments of *PpDDC-H181L*, *PpDDC-H181L-G344S*, *PpDDC-Y79F-F80Y-H181N* and *PpDDC-Y79F-F80Y-H181N-G344S* were PCR amplified from subcloning vectors and then cloned into pCDFDuet-1 digested with NcoI and BamHI via Gibson assembly. To produce pTYB21-PpDDC-S, *PpDDC-H181L* was PCR amplified and cloned into pTYB21 digested with SapI and BamHI via Gibson assembly. A *CjNCS* DNA fragment was obtained from NdeI and XhoI digestion of the *CjNCS* subcloning vector, and then cloned into pCDFDuet-1-PpDDC vectors via NdeI and XhoI sites to produce pCDFD-CjNCS-PpDDC. A *S. cerevisiae ARO10* gene fragment was digested with NcoI and NotI in the *ARO10* subcloning vector and then cloned into pCDFDuet-1 via NcoI and NotI restriction sites. A *CjNCS* gene fragment was next digested from the subcloning vector using NdeI and XhoI, and then cloned into pCDFDuet-1-ARO10 via the NdeI and XhoI restriction sites to produce pCDFD-CjNCS-ARO10. *E. coli HpaBC* containing gene fragments were PCR amplified from *E. coli* using the Gibson assembly primers shown in Supplementary Table 8. The PCR product was cleaned using a conventional column-based kit, and then cloned into XhoI-digested pET23 via Gibson assembly to produce pET23-EcHpaBC.

### In vivo production of BIA

BL21(DE3) and BL21-AI competent *E. coli* cells were transformed with various combinations of plasmids from Supplementary Table 7, resulting in the strains shown in Supplementary Table 2. Strains were tested in M9, LB or TB, supplemented with various substrates according to Supplementary Table 2. Expression of recombinant genes in expression vectors containing the T7 promoter system was induced by the addition of 0.5–1.5 mM isopropyl β-D-1-thiogalactopyranoside (IPTG) to BL21(DE3) cultures. When using BL21-AI cells 0.08–0.4% arabinose was included. Expression of PsPDC1, PsPDC2, and Ps2HCLL in pBAD-DEST49 was also induced by the addition of 0.08–0.4% arabinose.

For quantification of aromatic products, A1-01-DE3 (3 CjMTs, PsNMCH, CjNCS and ARO10), P1-02-AI (3CjMTs, PpDDC, PsONCS3 and PsPDC1), P1-04-AI (3CjMTs, PpDDC, PsONCS3, PsTyDC1 and PsPDC1), P1-06-DE3 (3CjMTs, PpDDC-Y79F-F80Y-H181N, PsONCS3 and PsPDC1), P1-07-AI (3CjMTs, PpDDC-Y79F-F80Y-H181N, PsONCS3 and PsPDC1), A1-06-AI (3 CjMTs, PpDDC-Y79F-F80Y-H181N, CjNCS, ARO10 and PsTyDC1) and T1-10-DE3 (3CjMTs, PpDDC, PsONCS3 and PsTyDC1) (Figs. 6–8, Supplementary Table 2, and Supplementary Fig. 5b, c) were grown using 3.5 mL teriffic broth (TB) supplemented with sodium ascorbate and appropriate antibiotics, in plastic culture tubes at 34–37 °C with shaking at 180–190 rpm. After reaching late log phase, inducing agent (IPTG or arabinose) and substrates (>8 mM tyrosine, >8 mM L-DOPA, >9 mM tyrosine-^13^*C*, >3 mM tyrosine-*d*_4_, >11 mM L-DOPA-*d*_3_) were added. When tyrosine was used as a substrate, sometimes dopamine was included as indicated in Supplementary Table 2 and Supplementary Fig 5b (4.7–7.5 mM dopamine, 10.3 mM dopamine-*d*2). The addition of dopamine together with L-DOPA was also tested with strain A1-01-DE3 as indicated in Supplementary Table 2 and Supplementary Fig. 5c (17.3 mM dopamine, 7.9 mM dopamine-*d*2). Cultures were then incubated at 25 °C with shaking at 180–200 rpm.

DT-01-DE3 (3CjMTs, PpDDC-Y79F-F80Y-H181N and TfNCS), DS-02-DE3 (3CjMTs, PsNMCH, CjNCS and PpDDC-H181L), DD-01-DE3 (3CjMTs, PsNMCH, CjNCS and PpDDC-H181L-G344S), DQ-01-DE3 (3CjMTs, PsNMCH, CjNCS and PpDDC-Y79F-F80Y-H181N-G344S), DT-02-DE3 (3CjMTs, PpDDC-Y79F-F80Y-H181N and PsONCS3), DT-03-DE3 (3CjMTs, PsNMCH, CjNCS and PpDDC-Y79F-F80Y-H181N) and A1-03-DE3 (3CjMTs, PpDDC, CjNCS, ARO10 and EcHpaBC) (Fig. 7a, Supplementary Table 2, and Supplementary Fig. 5a, d) were tested in 3–4.8 mL M9 supplemented with ascorbate and appropriate antibiotics. After reaching log phase in plastic culture tubes at 36–37 °C, IPTG and substrates (>4.5 mM tyrosine, >2 mM L-DOPA, >5 mM tyrosine-^13^*C*, >4 mM L-DOPA-*d*_3_) were added. When tyrosine was used as a substrate, sometimes dopamine was included as indicated in Supplementary Table 2 (1.2–1.4 mM dopamine). Cultures were then incubated at 20–25 °C with shaking at 180 rpm. Additional ascorbate was added as needed to prevent oxidative degradation of target compounds and melanization.

Conversion of norcoclaurine to reticuline was mediated by NMCH and CPR containing strains N1-01-DE3, N1-02-DE3, N1-03-DE3, N1-04-DE3, N2-01-DE3, N2-02-DE3, N2-03-DE3 and N2-04-DE3 (Supplementary Table 2). Here, strains first grown in LB medium were used to inoculate TB medium to a starting OD_600_ of 0.02 in 3 mL, with appropriate antibiotics. After four hours at 37 °C with shaking at 200 rpm, recombinant protein expression was induced with 0.68 mM IPTG and the temperature was lowered to 20 °C. After 5.5 h, cells were spun down and re-suspended in 1.5 mL TB supplemented with 1.2 mM norcoclaurine, 5.1 mM sodium ascorbate, and 0.2 mM IPTG. After 1.5 days at 25 °C with shaking at 200 rpm, BIA titers were measured with LC-MS.

Additional bioproduction conditions are given in the legends of Fig. 2e, Fig. 4e, Fig. 8a, Supplementary Fig. 2, Supplementary Fig. 3 and Supplementary Fig. 5. Bioproduction times are based on the addition of substrate.

### Quantitative analysis of BIA pathway intermediates with LC-MS, CE-MS, and GC-MS

The culture medium was filtered with Amicon Ultra 0.5 mL centrifugal filters with a molecular weight cut-off of 3000 Da. Filtrates were kept on ice and immediately processed for analysis, or stored at −30 °C or −80 °C before use.

For LC-MS analysis, filtered culture medium was diluted in a solution of camphor sulfonic acid, and then loaded onto a Shimadzu LCMS-8050 system (Shimadzu, Kyoto, Japan) operated in multiple reaction monitoring (MRM) mode^7^. The electrospray ionization (ESI) ion source was connected to a Shimadzu Nexera X2 UHPLC system where separation was performed on a Discovery HS F5-3 column (3 μm, 2.1 mm × 150 mm, Sigma–Aldrich). Shimadzu LabSolutions LCMS version 5.99 SP2 was used for data collection and analysis. DHPAA [151.30 > 123.15(−)], tyramine [138.00 > 121.15(+)], dopamine [154.10 > 91.05(+)], norcoclaurine [272.00 > 106.95(+)], norlaudanosoline [288.05 > 164.15(+)] and reticuline [330.10 > 192.00(+)] were identified using the MRM transitions listed in brackets, and confirmed by running authentic standards. Over 100 metabolites could be monitored with MRM detection.

For CE-MS analysis, filtered samples were diluted in a methionine sulfone solution when using positive ion mode, or in a piperazine-*N*,*N*’-bis(2-ethanesulfonic acid) solution for negative ion mode. CE-MS analysis was performed using an Agilent G7100 CE system with an Agilent G6224AA LC/MSD TOF (Agilent Technologies, Palo Alto, CA)^36,37^. Agilent MassHunter Workstation versions 10.1 and B.06.00 were used for data acquisition and analysis, respectively. Quantification of isotopes in Fig. 8 and Supplementary Fig. 5 was based on standard curves of non-labeled compounds. CE-MS peak areas in relation to internal standard peak areas were used to quantify all compounds except for 4HPP (Supplementary Fig. 5a), which was quantified based on its own peak intensity.

For GC–MS analysis of in vivo products, filtered samples were dried under vacuum and then derivatized with BSTFA and TMS-Cl. The derivatized aromatic compounds were analyzed on a GCMS-QP2010 Plus (Shimadzu) with a DB-5 capillary column (Agilent). Shimadzu LabSolutions version 2.72 was used for GC-MS data collection and analysis. TMS-derivatized tyrosol and norcoclaurine were identified using the most intense product ions *m/z* 179.1 and *m/z* 308.1, respectively, and confirmed by running authentic standards.

### In vitro characterizations of PsTyDC6

PsTyDC6 was expressed in Rosetta-gami 2 cells transformed with pTYB21-PsTyDC6 (Supplementary Table 7). After reaching log phase, the cells were induced with 0.15 mM IPTG and grown overnight at 15.5 °C. PsTyDC6 was purified on a chitin column followed by on-column cleavage of the chitin-binding domain and intein fusion via the addition of 50 mM DTT to the column. PsTyDC6 was then eluted into Amicon Ultra centrifugal filters and the buffer was changed to PBS (pH 7.0).

For detection of in vitro produced 4HPAA, purified PsTyDC6 and digested PsTyDC6 cell extract were mixed with 5 mM and 4 mM tyrosine, respectively. PsTyDC6 reactions containing 100 μM PLP were started together with control reactions containing 100 μM PLP and 4 mM tyrosine, followed by incubation at 30 °C for 3.5 h. Samples were lyophilized and then derivatized by treatment with a pyridine and methoxyamine solution followed by treatment with MSTFA. Derivatized compounds were analyzed by GC-MS. TMS- and methoxyamine-derivatized 4HPAA was identified based on product ions *m/z* 190.1 and *m/z* 205.1, and confirmed by running an authentic 4HPAA standard after derivatization using the same method. To detect in vitro production of H_2_O_2_ by PsTyDC6, a horseradish peroxidase-based fluorescent assay^7^ was performed with the fluorescent substrate Amplex Red (10-acetyl-3,7-dihydroxyphenoxazine) together with other components of a Fluorimetric Hydrogen Peroxide Assay Kit (Sigma-Aldrich). For the peroxidase-based assay, PsTyDC6 was prepared in PBS (pH 7.0) with 1 μM PLP. Baseline fluorescence from the control with matching PsTyDC6 and PLP, but with no tyrosine, was subtracted from each tested condition containing tyrosine. Initial rates of fluorescence production were plotted against final tyrosine concentration using the Michaelis-Menten function of Prism 7 version 7.0d.

LC-MS operated in MRM mode was applied to detect in vitro produced DHPAA, dopamine, tyramine, norcoclaurine and norlaudanosoline. For DHPAA, dopamine and norlaudanosoline production, purified PsTyDC6 was mixed with 5 mM L-DOPA. For tyramine and norcoclaurine production, purified PsTyDC6 was mixed with 1.25 mM tyrosine and 2.5 mM L-DOPA. In vitro samples were incubated at 30 °C for 80 min. to analyze DHPAA, and for 8 h to analyze norcoclaurine and norlaudanosoline.

### Extraction of aromatic compounds for GC-MS quantification

A solution of ammonium carbonate was added to culture samples, followed by addition of ethyl acetate. After vortexing, the organic layer was removed and evaporated under vacuum. The dried extracts were then derivatized in a mixture of BSTFA, TMS-Cl, and ethyl acetate. Quantitative standard curves were produced by extracting alkaloid standards prepared in TB medium, followed by TMS-derivatization in equivalent volumes. The TMS-derivatized samples were analyzed with GC-MS as described above.

### Reporting summary

Further information on research design is available in the Nature Research Reporting Summary linked to this article.

## Supplementary information

Supplementary Information

Description of Additional Supplementary Files

Supplementary Data 1

Supplementary Data 2

Supplementary Data 3

Supplementary Data 4

Supplementary Data 5

Supplementary Data 6

Supplementary Data 7

Supplementary Data 8

Reporting Summary

## Data Availability

Data supporting the findings of this work are available within the paper and its Supplementary Information files. Training sequences and information for machine learning models are included as Supplementary Data files. A reporting summary for this Article is available as a Supplementary Information file. All protein accessions used in this study are available from the National Center for Biotechnology Information (NCBI) database [https://www.ncbi.nlm.nih.gov]. Protein Data Bank (PDB) structures including 2Q5O and 2WVA are available from the PDB database. Source data are provided with this paper.

## Source data

Source Data

## Code availability

The computer code used for SVM prediction of enzyme sequences is available on GitHub [https://github.com/nwatanbe/SVM_E_model].
