# Supplementary material for: Machine learning discovery of missing links that mediate alternative branches to plant alkaloids
Source: Nat Commun. 2022 Mar 16;13:1405. doi: 10.1038/s41467-022-28883-8 (PMC8927377; doi:10.1038/s41467-022-28883-8)
Supplement: Supplementary file 1 — Supplementary Information [file 41467_2022_28883_MOESM1_ESM.pdf]

Supplementary Information for

Machine learning discovery of missing links that mediate alternative branches to plant alkaloids

Vavricka *et al.*

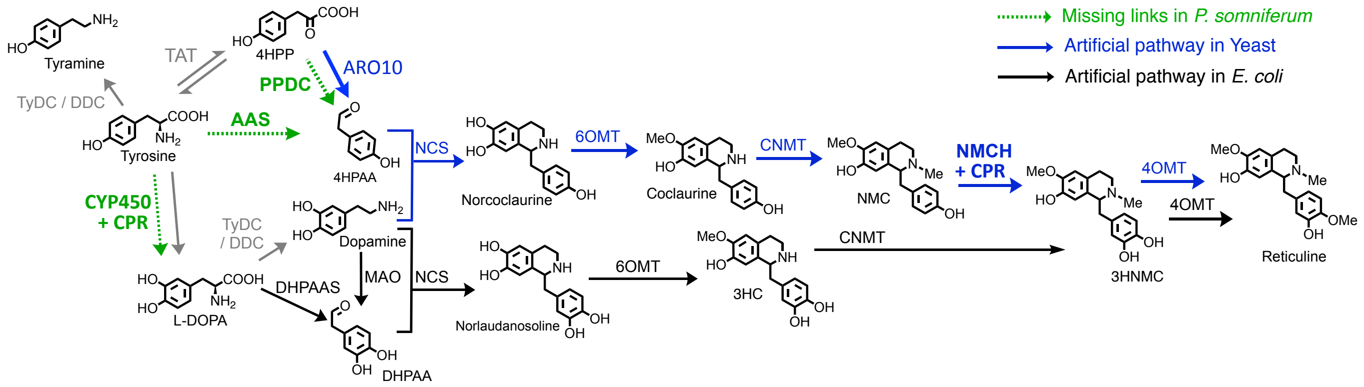

Supplementary Figure 1 | Comparison of natural and engineered benzylisoquinoline pathways. Steps mediated by unclear *P. somniferum* enzymes are shown as green dotted arrows. Reconstructed pathways to reticuline in yeast and *E. coli* are shown with blue or black arrows, respectively. Metabolite abbreviations: 4HPP - 4-hydroxyphenylpyruvic acid, 4HPAA - 4-hydroxyphenylacetaldehyde, L-DOPA - 3,4-dihydroxy-L-phenylalanine, DHPAA - 3,4-dihydroxyphenylacetaldehyde, NMC - *N*-methylcoclaurine, 3HC - 3-hydroxycoclaurine, 3HNMC 3-hydroxy-*N*-methylcoclaurine. Enzyme abbreviations: AAS - aromatic acetaldehyde synthase, DHPAAS - 3,4-dihydroxyphenylacetaldehyde synthase, PPDC - phenylpyruvate decarboxylase, TAT - L-tyrosine aminotransferase, TyDC - L-tyrosine decarboxylase, DDC - L-DOPA decarboxylase, CYP450 - cytochrome P450, CPR - CYP450 reductase, ARO10 - *Saccharomyces cerevisiae* transaminated amino acid decarboxylase, NCS - norcoclaurine synthase, 6OMT - norcoclaurine 6-*O*-methyltransferase, CNMT - coclaurine *N*-methyltransferase, NMCH - *N*-methylcoclaurine 3-hydroxylase, 4OMT - 3-hydroxy-*N*-methylcoclaurine 4-*O*-methyltransferase.

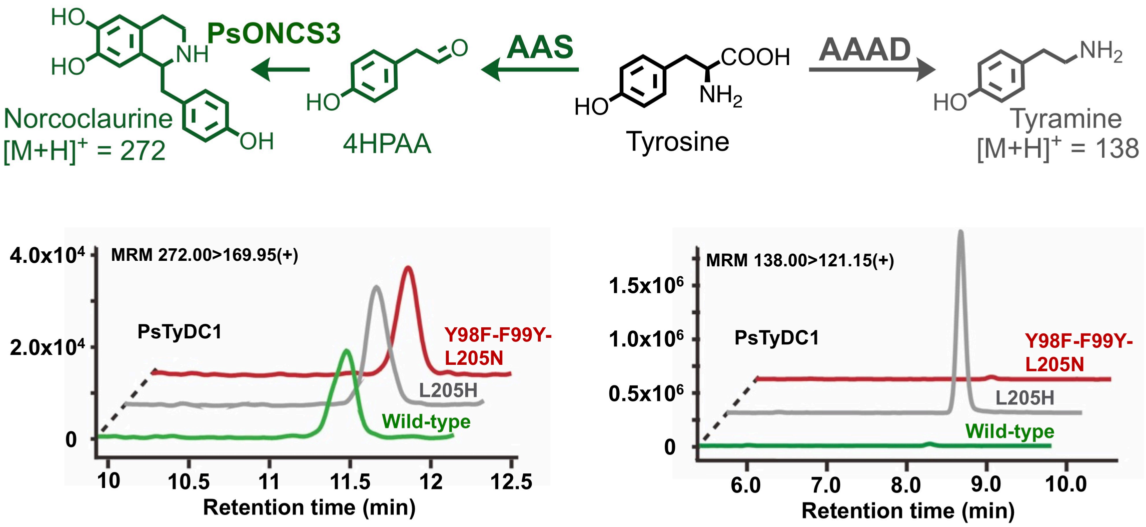

Supplementary Figure 2 | LC-MS detection of AAS and AAAD products from PsONCS3 containing strains. Strains T1-04-DE3 (wild-type PsTyDC1 and PsONCS3), T1-05-DE3 (PsTyDC1-L205H and PsONCS3) and T1-06-DE3 (PsTyDC1-Y98F-F99Y-L205N and PsONCS3) were grown in LB supplemented with 1 mM tyrosine and 0.5 mM dopamine, at 28°C with 180 rpm shaking for 51 hours. Here, norcoclaurine production is generally lower with codon optimized PsONCS3, than it is with codon optimized TfNCS shown in Fig. 2e.

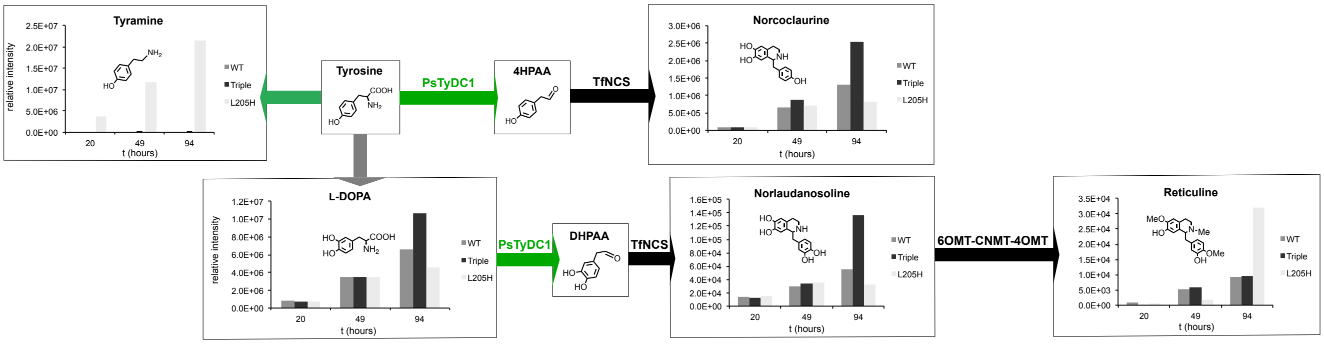

Supplementary Figure 3 | LC-MS analysis of hybrid pathway intermediates resulting from PsTyDC1, PsNMCH, TfNCS and three *Coptis japonica* BIA methyltransferases (6OMT, CNMT and 4OMT). Here, it is assumed that PsNMCH is not functioning without expression of CYP450 reductase (CPR). Strains T1-07-DE3 (wild-type PsTyDC1), T1-09-DE3 (PsTyDC1-Y98F-F99Y-L205N, labeled as "Triple") and T1-08-DE3 (PsTyDC1-L205H) were grown in M9 (n=2). Recombinant protein expression was induced during log phase with 0.8 mM IPTG, and 0.5 hours later 5 mM tyrosine and 2.5 mM dopamine were added. BIA production at 20-25°C with 180 rpm shaking was monitored over 94 hours.

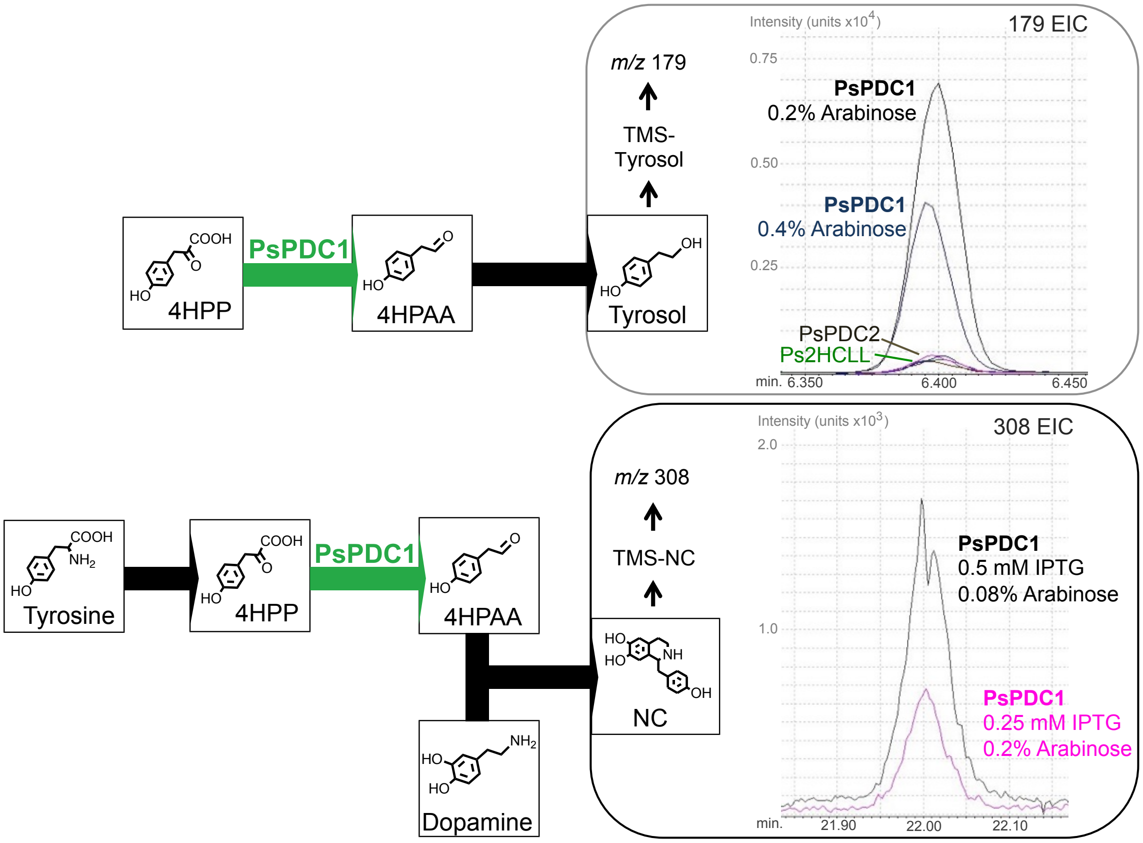

Supplementary Figure 4 | Replicated PsPDC1 mediated production of tyrosol and norcoclaurine. In vivo tyrosol is detected from conversion of 4-hydroxyphenylpyruvic acid (4HPP) by strains P1-01-AI (PsPDC1), P2-01-AI (PsPDC2) and P3-01-AI (Ps2HCLL), as described for Fig. 4e. In vivo norcoclaurine (NC) is produced from tyrosine and dopamine by strain P1-02-AI (PsPDC1, PpDDC, PsONCS3, Cj6OMT, CjCNMT and Cj4OMT), as described for Fig. 4e.

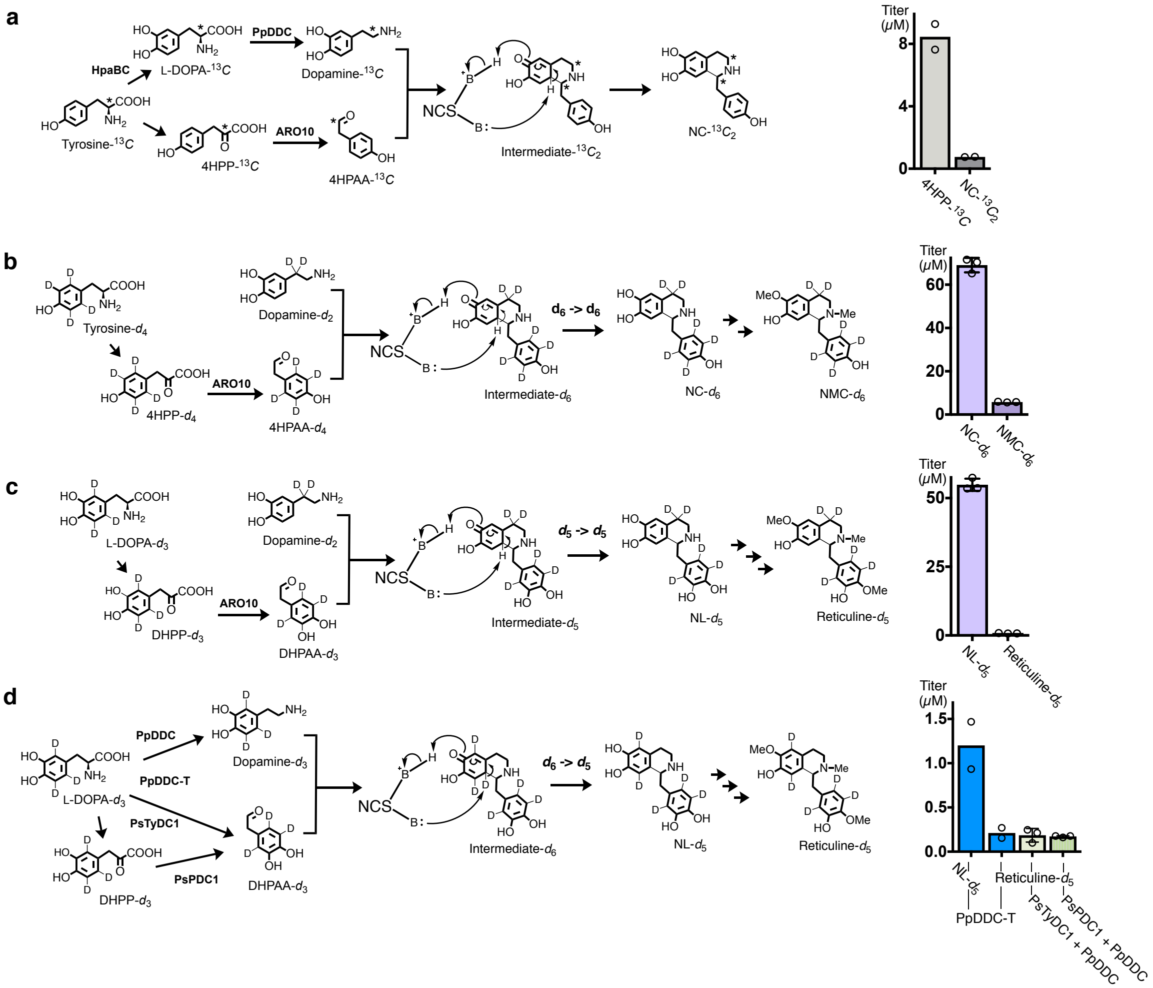

Supplementary Figure 5 | Isotope profiling of synthetic BIA pathways. NCS-mediated production of $^{13}C_2$-labeled (**a**), d_6-labeled (**b**) and d_5-labeled (**c**, **d**) BIAs, based on the proposed mechanism of TfNCS[38,39]. **a**, Metabolism of tyrosine-^{13}C by strain A1-03-DE3 (Supplementary Table 2) in M9 medium. Production of NC-$^{13}C_2$ (day 7, n=2) confirms the PPDC bypass through 4HPP-^{13}C (day 3, n=2). **b**, Strain A1-01-DE3 produced NMC-d_6 from tyrosine-d_4 and dopamine-d_2 in TB (t = 61 hours, n=3). **c**, A1-01-DE3 also produced reitculine-d_5 from L-DOPA-d_3 and dopamine-d_2 in TB (t = 61 hours, n=3), through a DHPP branch pathway. **d**, NL-d_5 and reticuline-d_5 were produced by the PpDDC-Y79F-F80Y-H181N (PpDDC-T) containing strain DT-03-DE3 in M9 supplemented with L-DOPA-d_3, where the titers on day 7 are presented (n=2). Reticuline-d_5 production by strains T1-10-DE3 and P1-02-AI in TB is compared (n=3). Here, NCS catalyzes the loss of a deuterium from dopamine-d_3. SAM-dependent methyltransferase bottlenecks are indicated by **b**-**d**. Additional culture conditions are described in the methods section. Abbreviations: L-DOPA - 3,4-dihydroxy-L-phenylalanine, 4HPP - 4-hydroxyphenylpyruvic acid, 4HPAA - 4-hydroxyphenylacetaldehyde, NC - norcoclaurine, NMC - *N*-methylcoclaurine, DHPP - 3,4-dihydroxyphenylpyruvic acid, DHPAA - 3,4-dihydroxyphenylacetaldehyde, NL - norlaudanosoline. Individual samples were analyzed 2 or 3 times (n=2 or n=3) and plotted using Prism 7. Error bars represent mean values +/- standard deviations.

Supplementary Table 1 | High-dimensional SVM-based selection of *P. somniferum* sequences from AAS and AAAD prediction models

Aromatic acetaldehyde synthase (AAS) prediction model

Annotation	Accession	Positive P	Negative P	Decision
**PsTyDC1**	**P54768.1**	**0.96786**	**0.03214**	**0.84335**
PsTyDC2	XP_026405553.1	0.85375	0.14625	0.53173
PsTyDC3	P54770.2	0.88638	0.11362	0.58669
PsTyDC5	P54771.1	0.95300	0.04700	0.76816
**PsTyDC6**	**XP_026437934.1**	**0.97920**	**0.02080**	**0.92828**
PsTyDC7	AAC61843.1	0.75992	0.24008	0.41567
PsTyDC8	AAC61841.1	0.85300	0.14700	0.53059

Typical aromatic amino acid decarboxylase (AAAD) prediction model

Annotation	Accession	Positive P	Negative P	Decision
**PsTyDC1**	**P54768.1**	**0.98861**	**0.01139**	**0.58724**
PsTyDC2	XP_026405553.1	0.93583	0.06417	0.30054
PsTyDC3	P54770.2	0.94825	0.05175	0.33725
PsTyDC5	P54771.1	0.92130	0.07870	0.26521
**PsTyDC6**	**XP_026437934.1**	**0.99300**	**0.00700**	**0.66613**
PsTyDC7	AAC61843.1	0.92177	0.07823	0.26626
PsTyDC8	AAC61841.1	0.99998	0.00002	0.83410

P. somniferum TyDC4 (PsTyDC4) has a premature stop codon, and is not included. "Positive P" is positive probability and "Negative P" is negative probability. Decision scores represent the distance from the SVM prediction boundary. PsTyDC6 and PsTyDC1, shown in bold font, score high for AAS activity and are tested in the current study.

Supplementary Table 2 | Aromatic producing strains of this study

Strain	Genotype	Conditions	Products
BL21(DE3)	F– ompT gal dcm lon hsdSB(rB–mB–) λ(DE3 [lacI lacUV5-T7p07 ind1 sam7 nin5]) [malB+]K-12(λS)	-	-
BL21-AI	F– ompT gal dcm lon hsdSB(rB–mB–) [malB+]K-12(λS) araB::T7RNAP-tetA	-	-
Rosetta-gami 2(DE3)	Δ(ara-leu)7697 ΔlacX74 ΔphoA PvuII phoR araD139 ahpC galE galK rpsL (DE3) F′[lac+ lacIq pro] gor522::Tn10 trxB pRARE2 (CamR, StrR, TetR)	-	-
T1-01-DE3	pCDFD-TfNCS-PsTyDC1	LB-Tyr+DA	NC
T1-02-DE3	pCDFD-TfNCS-PsTyDC1-S	LB-Tyr+DA	Tyramine
T1-03-DE3	pCDFD-TfNCS-PsTyDC1-T	LB-Tyr+DA	NC
T1-04-DE3	pCDFD-PsONCS3-PsTyDC1	LB-Tyr+DA	trace NC
T1-05-DE3	pCDFD-PsONCS3-PsTyDC1-S	LB-Tyr+DA	Tyramine
T1-06-DE3	pCDFD-PsONCS3-PsTyDC1-T	LB-Tyr+DA	trace NC
T1-07-DE3	pACYC-3CjMTs-PsNMCH, pCDFD-TfNCS-PsTyDC1	M9-Tyr+DA	Reticuline
T1-08-DE3	pACYC-3CjMTs-PsNMCH, pCDFD-TfNCS-PsTyDC1-S	M9-Tyr+DA	Reticuline
T1-09-DE3	pACYC-3CjMTs-PsNMCH, pCDFD-TfNCS-PsTyDC1-T	M9-Tyr+DA	Reticuline
T1-10-DE3	pACYC-3CjMTs-PpDDC, pCDFD-PsONCS3-PsTyDC1	TB-DOPA	1 μM Reticuline
T1-11-DE3	pTYB21-PsTyDC1	-	-
T1-01-ROS	pTYB21-PsTyDC1	-	-
T6-01-DE3	pTYB21-PsTyDC6	-	-
T6-01-ROS	pTYB21-PsTyDC6	-	-
P1-01-AI	pBAD-PsPDC1	M9-4HPP	Tyrosol
P2-01-AI	pBAD-PsPDC2	M9-4HPP	
P3-01-AI	pBAD-Ps2HCLL	M9-4HPP	
P1-02-AI	pACYC-3CjMTs-PpDDC, pCDFD-PsONCS3, pBAD-PsPDC1	M9-Tyr+DA; **TB-DOPA**	NC; **1.5 μM Reticuline**
P1-03-DE3	pACYC-3CjMTs-PsNMCH, pCDFD-PsONCS3-PsTyDC1, pBAD-PsPDC1	TB-Tyr+DA	NC
P1-04-AI	pACYC-3CjMTs-PpDDC, pCDFD-PsONCS3-PsTyDC1, pBAD-PsPDC1	TB-DOPA	1.5 μM Reticuline
N1-01-DE3	pET23a-3PsMTs, pCOLAD-PsNMCH-PsCPR-L	TB-NC	27.8 μM Reticuline
N1-02-DE3	pET23a-3PsMTs, pCOLAD-PsNMCH-H203Y-PsCPR-L	TB-NC	15.8 μM Reticuline
N1-03-DE3	pET23a-3PsMTs, pCOLAD-PsNMCH-AtATR2	TB-NC	15.9 μM Reticuline
N1-04-DE3	pET23a-3PsMTs, pCOLAD-PsNMCH-H203Y-AtATR2	TB-NC	8.0 μM Reticuline
N2-01-DE3	pET23a-3PsMTs, pCOLAD-EcNMCH-AtATR2	TB-NC	4.9 μM Reticuline
N2-02-DE3	pET23a-3PsMTs, pCOLAD-EcNMCH-Y202H-AtATR2	TB-NC	8.4 μM Reticuline
N2-03-DE3	pET23a-3PsMTs, pCOLAD-EcNMCH-PsCPR-L	TB-NC	3.7 μM Reticuline
N2-04-DE3	pET23a-3PsMTs, pCOLAD-EcNMCH-Y202H-PsCPR-L	TB-NC	3.6 μM Reticuline
DS-01-DE3	pACYC-3CjMTs-PpDDC-S, pCDFD-PsONCS3	M9-DOPA	
DT-01-DE3	pACYC-3CjMTs-PpDDC-T, pCDFD-TfNCS	M9-DOPA	16.8 μM NL, 2.5 μM Reticuline
DT-02-DE3	pACYC-3CjMTs-PpDDC-T, pCDFD-PsONCS3	M9-DOPA	34 μM NL, 6.4 μM Reticuline
DS-02-DE3	pACYC-3CjMTs-PsNMCH, pCDFD-CjNCS-PpDDC-S	M9-Tyr+DA	NC
DD-01-DE3	pACYC-3CjMTs-PsNMCH, pCDFD-CjNCS-PpDDC-D	M9-Tyr+DA	
DT-03-DE3	pACYC-3CjMTs-PsNMCH, pCDFD-CjNCS-PpDDC-T	M9-DOPA	1.4 μM NL
DQ-01-DE3	pACYC-3CjMTs-PsNMCH, pCDFD-CjNCS-PpDDC-Q	M9-Tyr+DA	
DS-03-DE3	pACYC-3CjMTs-PpDDC, pCDFD-CjNCS-PpDDC-S, pET23a-EcHpaBC	M9-Tyr	NC
DD-02-DE3	pACYC-3CjMTs-PpDDC, pCDFD-CjNCS-PpDDC-D, pET23a-EcHpaBC	M9-Tyr	
DT-04-DE3	pACYC-3CjMTs-PsNMCH, pCDFD-CjNCS-PpDDC-T, pET23a-EcHpaBC	M9-Tyr	DA, NL
T1-12-DE3	pACYC-3CjMTs-PpDDC, pCDFD-PsONCS3-PsTyDC1, pET23a-EcHpaBC	LB-Tyr	DA
A1-01-DE3	pACYC-3CjMTs-PsNMCH, pCDFD-CjNCS-ARO10	**TB-Tyr+DA**; TB-DOPA+DA	**356 μM NC**, **240 mM NMC**; 1 μM Reticuline
A1-02-DE3	pACYC-3CjMTs-PpDDC, pCDFD-CjNCS-ARO10		
A1-03-DE3	pACYC-3CjMTs-PpDDC, pCDFD-CjNCS-ARO10, pET23a-EcHpaBC	M9-Tyr	NC
DS-04-DE3	pACYC-3CjMTs-PpDDC, pCDFD-CjNCS-PpDDC-S, pET23a-EcHpaBC, pE-DHPAAS		
A1-05-DE3	pACYC-3CjMTs-PsNMCH, pCDFD-CjNCS-ARO10, pTrc-DHPAAS-T	TB-Tyr+DOPA	
A1-06-AI	pACYC-3CjMTs-PpDDC-T, pCDFD-CjNCS-ARO10, pTXB1-PsTyDC1	TB-Tyr+DOPA; **TB-Tyr+DA**	74.9 μM Reticuline; **112 μM NMC**
P1-05-AI	pACYC-3CjMTs-PsNMCH, pCDFD-CjNCS-PpDDC-T, pBAD-PsPDC1	TB-Tyr+DOPA	
P1-06-DE3	pACYC-3CjMTs-PpDDC-T, pCDFD-PsONCS3, pBAD-PsPDC1	TB-Tyr+DOPA	3.7 μM Reticuline
P1-07-AI	pACYC-3CjMTs-PpDDC-T, pCDFD-PsONCS3, pBAD-PsPDC1	TB-Tyr+DOPA	61.8 μM Reticuline

Strains with names ending with "DE3" are derived from BL21(DE3), strains ending with "AI" are derived from BL21-AI, and strains ending with "ROS" are derived from Rosetta gami 2. Plasmid details are given in Supplementary Table 7. The last two columns list successfully tested in vivo

conditions (growth medium and added substrate) and aromatic products. Concentrations of extracted *N*-methylcoclaurine (NMC) and reticuline per culture volume are listed for AI-01-DE3, A1-06-AI, P1-06-DE3 and P1-07-AI; all other listed concentrations represent titers in filtered culture medium. Only product titers quantified at or above 1 μM are listed. Matched substrates and corresponding products are indicated by bold font. Abbreviations: S - single variant, D - double variant, T - triple variant, Q - quadruple variant, PsONCS3 - *P. somniferum* multi-domain NCS, CjNCS - *Coptis japonica* NCS, 3CjMTs - *C. japonica* 6OMT, CNMT and 4OMT, 3PsMTs - *P. somniferum* 6OMT, CNMT and 4OMT, PsPDC - *P. somniferum* pyruvate decarboxylase, Ps2HCLL - *P. somniferum* 2-hydroxyacyl-CoA ligase-like, PpDDC - *P. putida* L-DOPA decarboxylase, PsNMCH - *P. somniferum* *N*-methylcoclaurine 3-hydroxylase, PsCPR-L - *P. somniferum* CYP450 reductase-like, AtATR2 - *A. thalania* CYP450 reductase 2, EcHpaBC - *E. coli* 4-hydroxyphenylacetate 3-monooxygenase complex, ARO10 - *S. cerevisiae* phenylpyruvate decarboxylase, DHPAAS - *Bombyx mori* 3,4-dihyxroxyphenylacetaldehyde synthase, Tyr - tyrosine, 4HPP - 4-hydroxyphenylpyruvate, DA - dopamine, DOPA - 3,4-dihydroxy-L-phenylalanine, NC - norcoclaurine, NL - norlaudanosoline, NMC - *N*-methylcoclaurine.

Supplementary Table 3 | High-dimensional SVM-based prediction of *P. somniferum* sequences with potential PPDC activity

Combined PPDC and PDC prediction

Gene Annotation	Accession	Predict	Positive P	Negative P	Decision
**2-hydroxyacyl-CoA lyase-like**	**XP_026454385.1**	**1**	**0.92398**	**0.07602**	**0.48808**
2-hydroxyacyl-CoA lyase-like	XP_026441845.1	1	0.89096	0.10904	0.39427
**PsPDC1**	**XP_026414621.1***	**1**	**0.99999**	**6.37E-09**	**2.2575**
PsPDC1-like	8 Genes**	1	0.999 - 1	2.1E-10 - 2.1E-07	1.84 - 2.66
PsPDC1 isoform X1	XP_026435244.1	1	0.99999	1.13E-08	2.1894
**PsPDC2**	**XP_026435245.1**	**1**	**0.93910**	**0.06090**	**0.54435**
C5167_004675	RZC57375.1	1	0.99999	5.85E-08	1.9955
C5167_027778	RZC91716.1	1	0.99999	1.41E-06	1.6197
**TrcPsPDC1-IX1**	**RZC88315.1*****	**1**	**0.99999**	**1.25E-09**	**2.4504**
C5167_033954	RZC70805.1	1	0.99999	2.33E-06	1.5606
C5167_010599	RZC66910.1	0	0.00016	0.99984	-2.1727
C5167_008924	RZC65241.1	1	0.99999	1.54E-09	2.4250
C5167_025498	RZC63722.1	1	0.99537	0.00463	1.1670
C5167_006728	RZC59427.1	0	0.00047	0.99953	-1.9141
C5167_004675	RZC57375.1	1	0.99999	5.85E-08	1.9955
C5167_015702	RZC56842.1	1	0.99999	1.18E-09	2.4570
C5167_019208	RZC50776.1	0	0.02530	0.97470	-0.9697
C5167_018424	RZC50005.1	0	7.30E-06	0.99999	-2.8963
C5167_017103	RZC48677.1	1	0.99999	1.31E-06	1.6281
C5167_039607	RZC46647.1	1	0.99999	2.70E-09	2.3591

PPDC prediction

Gene Annotation	Accession	Predict	Positive P	Negative P	Decision
**2-hydroxyacyl-CoA lyase-like**	**XP_026454385.1**	**0**	**0.08090**	**0.91910**	**-0.46727**
2-hydroxyacyl-CoA lyase-like	XP_026441845.1	0	0.05945	0.94055	-0.54148
**PsPDC1**	**XP_026414621.1***	**1**	**0.64111**	**0.35889**	**0.12354**
PsPDC1-like	8 Genes**	0-1	0.45 - 0.98	0.02 - 0.054	-0.02 - 0.74
PsPDC1 isoform X1	XP_026435244.1	1	0.97835	0.02165	0.76317
**PsPDC2**	**XP_026435245.1**	**0**	**0.10512**	**0.89488**	**-0.41035**
C5167_004675	RZC57375.1	1	0.83452	0.16548	0.33077
C5167_027778	RZC91716.1	0	0.40677	0.59323	-0.06289
**TrcPsPDC1-IX1**	**RZC88315.1*****	**1**	**0.99012**	**0.00988**	**0.92032**
C5167_033954	RZC70805.1	1	0.66538	0.33462	0.14741
C5167_010599	RZC66910.1	0	0.00002	0.99998	-2.16718
C5167_008924	RZC65241.1	1	0.97997	0.02003	0.77884
C5167_025498	RZC63722.1	0	0.19233	0.80767	-0.27103
C5167_006728	RZC59427.1	0	0.00000	1.00000	-2.78298
C5167_004675	RZC57375.1	1	0.83452	0.16548	0.33077
C5167_015702	RZC56842.1	1	0.82369	0.17631	0.31574
C5167_019208	RZC50776.1	0	0.00050	0.99950	-1.48492
C5167_018424	RZC50005.1	0	0.00000	1.00000	-2.61536
C5167_017103	RZC48677.1	1	0.90238	0.09762	0.45003
C5167_039607	RZC46647.1	1	0.97113	0.02887	0.70494

Sequences shown in bold font were tested in this study. The above combined PPDC and PDC model is trained with positive sequences that include typical PDC sequences (Supplementary Data 3). The lower PPDC prediction model is trained with positive training sequences annotated as phenylpyruvate decarboxylase and indolepyruvate decarboxylase, plus rose PPDC (BAU70033.1) and 19 sequences phylogenetically related to rose PPDC (Supplementary Data 4). All "C5167" sequences are annotated as "hypothetical protein"; *PsPDC1 = XP_026414621.1/RZC79432.1; **PsPDC1-like = XP_026411619.1/RZC72691.1, XP_026392057.1, XP_026379022.1, XP_026379014.1, XP_026458198.1,

XP_026425556.1/RZC84511.1, XP_026412580.1, XP_026441200.1; C5167_015702 - 91.09% to PsPDC1, C5167_004675 - 90.28% to PsPDC1; C5167_008924 - 97.84% to PsPDC1-like (XP_026392057.1); C5167_039607 - 98.17% to PsPDC1-like (XP_026412580.1). ***RZC88315.1 is annotated as C5167_016119, is 100% identical to PsPDC1 isoform X1 with a 27 residue N-terminal truncation, and is herein referred to as truncated PsPDC1 isoform X1 (TrcPsPDC1-IX1).

Supplementary Table 4 | High-dimensional SVM-based prediction of plant CYP450 sequences with potential NMCH activity

NMCH prediction					
Annotation	Accession	Predict	Positive P	Negative P	Decision
**EcNMCH**	**O64899**	**1**	**0.99998**	**0.00002**	**1.17088**
**EcNMCH-Y202H**	**-**	**1**	**0.99999**	**0.00001**	**1.21161**
**PsNMCH-I1**	**XP_026418925.1**	**1**	**0.99734**	**0.00266**	**1.15617**
**PsNMCH-H203Y**	**-**	**1**	**0.99691**	**0.00309**	**1.12749**
NMCH, partial	ACM44071.1	0	0.00000	1.00000	-2.50698
NMCH-I1	Q9SP06.1	1	0.99630	0.00370	1.09338
NMCH, partial	AAF61400.1	1	0.99571	0.00429	1.06542
NMCH-I1-like	XP_026389058.1	1	0.99682	0.00318	1.12202
C5167_025060	RZC63317.1	0	0.00003	0.99997	-1.91714
NMCH-like	XP_026431349.1	1	0.99205	0.00795	0.94750
NMCH-like	I3V6B1.1	1	0.94564	0.05436	0.57310
G8HL	XP_026380180.1	1	0.95818	0.04182	0.62544
G8HL	XP_026411599.1	1	0.98144	0.01856	0.78437
G8HL	XP_026380181.1	1	0.46856	0.53144	0.00719
G8HL	XP_026409442.1	1	0.99585	0.00415	1.07140
C5167_048514	RZC73039.1	1	0.99092	0.00908	0.92188
G8HL	XP_026379063.1	0	0.27006	0.72994	-0.15766
G8HL	XP_026380179.1	1	0.96829	0.03171	0.68003
C5167_004855	RZC57552.1	0	0.00702	0.99298	-0.91017
G8HL	XP_026409440.1	0	0.21098	0.78902	-0.21931
G8HL	XP_026409437.1	1	0.96278	0.03722	0.64849
G8HL	XP_026379135.1	1	0.91573	0.08427	0.48369
G8HL	XP_026377890.1	0	0.10806	0.89194	-0.36960
CYP71A1-like	XP_026430038.1	1	0.86340	0.13660	0.38092
C5167_026704	RZC86034.1	1	0.96577	0.03423	0.66503
F3ML	XP_026441287.1	1	0.91960	0.08040	0.49340
C5167_027831	RZC91763.1	1	0.87236	0.12764	0.39573
CYP76A2-like	XP_026447672.1	1	0.67565	0.32435	0.17042
CYP76A2-like	XP_026447278.1	1	0.59133	0.40867	0.10077
F3ML	XP_026381164.1	0	0.19259	0.80741	-0.24100
C5167_005813	RZC58514.1	0	0.01516	0.98484	-0.76399
CYP450	AFK73718.1	0	0.02419	0.97581	-0.67490
CYP76A2-like	XP_026450500.1	0	0.28013	0.71987	-0.14808
C5167_002759	RZC76181.1	0	0.05990	0.94010	-0.50025
DHP6AML	XP_026442746.1	0	0.04346	0.95654	-0.56258
G8HL	XP_026411431.1	0	0.05814	0.94186	-0.50609
F3ML	XP_026427767.1	0	0.08272	0.91728	-0.42565
F3ML	XP_026418653.1	0	0.00994	0.99006	-0.84420
CYP736A12-like	XP_026430405.1	1	0.80286	0.19714	0.29765
CYP736A12-like	XP_026437612.1	1	0.85694	0.14306	0.37074
CYP76A2-like	XP_026437513.1	0	0.00554	0.99446	-0.95524
CYP76A1-like	XP_026453156.1	1	0.85751	0.14249	0.37162
CYP76A1-like	XP_026451658.1	0	0.44409	0.55591	-0.01159
G8HL	XP_026380438.1	0	0.03817	0.96183	-0.58759
G8HL	XP_026380437.1	0	0.03139	0.96861	-0.62511
G8HL	XP_026380436.1	0	0.02108	0.97892	-0.70119
C5167_048523, partial	RZC73046.1	0	0.35366	0.64634	-0.08523
CYP736A12-like	XP_026459019.1	0	0.10446	0.89554	-0.37680
ECODL	XP_026408386.1	0	0.06783	0.93217	-0.46638
G8HL	XP_026396971.1	0	0.20264	0.79736	-0.22896
CYP736A12-like	XP_026388372.1	1	0.63926	0.36074	0.13721
CYP76A2-like	XP_026451160.1	0	0.02247	0.97753	-0.68904
FSL isoform X1	XP_026380444.1	0	0.01065	0.98935	-0.83109
CYP76A1-like	XP_026399590.1	0	0.18332	0.81668	-0.25253
C5167_033939	RZC70799.1	0	0.12354	0.87646	-0.34086
CYP450	AFK73714.1	0	0.19020	0.80980	-0.24393
G8HL	XP_026393993.1	0	0.25886	0.74114	-0.16860
C5167_013475	RZC54568.1	0	0.05053	0.94947	-0.53339
CYP76C4-like	XP_026430487.1	0	0.03773	0.96227	-0.58982
CYP76C4-like	XP_026430486.1	0	0.02641	0.97359	-0.65815
CYP76A2-like	XP_026429624.1	0	0.20841	0.79159	-0.22226
CYP736A12-like	XP_026460423.1	0	0.33671	0.66329	-0.10030

C5167_044785	RZC90154.1	1	0.77720	0.22280	0.26828
CYP71A9-like	XP_026400228.1	0	0.10221	0.89779	-0.38141
CYP76C4-like	XP_026458250.1	0	0.01934	0.98066	-0.71757
C5167_015732	RZC56884.1	0	0.02417	0.97583	-0.67510
C5167_019753	RZC51326.1	1	0.80985	0.19015	0.30613
CYP736A12-like	XP_026437453.1	1	0.78432	0.21568	0.27617
CYP71A9-like	XP_026444276.1	0	0.02361	0.97639	-0.67957
C5167_027648	RZC91585.1	0	0.04574	0.95426	-0.55271
CYP71A9-like	XP_026378190.1	0	0.04165	0.95835	-0.57079
CYP736A12-like	XP_026388374.1	0	0.03185	0.96815	-0.62235
CYP71D8-like	XP_026451198.1	1	0.58274	0.41726	0.09419
CYP71A1-like	XP_026447646.1	0	0.08269	0.91731	-0.42572
CYP71A1-like isoform X1	XP_026380212.1	0	0.13900	0.86100	-0.31510
CYP736A12-like	XP_026460422.1	0	0.02623	0.97377	-0.65945
C5167_026541	RZC85882.1	0	0.18104	0.81896	-0.25543
C5167_005175	RZC57870.1	0	0.00000	1.00000	-4.11315
CYP71D8-like	XP_026392887.1	1	0.88125	0.11875	0.41133
C5167_009556	RZC65863.1	1	0.57069	0.42931	0.08497
CYP450	AFK73720.1	0	0.11183	0.88817	-0.36229
CYP71A1-like	XP_026377587.1	0	0.27175	0.72825	-0.15604
CYP76A2-like	XP_026443290.1	0	0.03203	0.96797	-0.62125
CYP71A9-like	XP_026458455.1	0	0.18324	0.81676	-0.25264
CYP76A2-like	XP_026440631.1	0	0.00248	0.99752	-1.10821
ECODL	XP_026410296.1	0	0.02352	0.97648	-0.68032
CYP98A2-like	XP_026458081.1	0	0.14007	0.85993	-0.31341
CYP98A2-like	XP_026403623.1	0	0.29674	0.70326	-0.13271
CYP71D8-like	XP_026407054.1	1	0.61615	0.38385	0.11970
PSOL	XP_026392886.1	1	0.80185	0.19815	0.29643
CYP76A2-like	XP_026440487.1	0	0.39833	0.60167	-0.04770
CYP76A2-like	XP_026429558.1	0	0.09799	0.90201	-0.39031
FSL	XP_026380449.1	0	0.03591	0.96409	-0.59934
CYP76AD1-like	XP_026380446.1	0	0.01865	0.98135	-0.72449
F3ML	XP_026380466.1	0	0.04601	0.95399	-0.55158
F3ML	XP_026410457.1	0	0.14052	0.85948	-0.31270
CYP71A1-like	XP_026409602.1	0	0.02340	0.97660	-0.68124
F35H1L	XP_026378021.1	1	0.53193	0.46807	0.05538
C5167_036448	RZC43501.1	0	0.01755	0.98245	-0.73613
CYP71D9-like	XP_026407073.1	1	0.65729	0.34271	0.15473
CYP76A2-like	XP_026429642.1	1	0.53654	0.46346	0.05888
C5167_026557	RZC85878.1	0	0.37591	0.62409	-0.06618
PSOL	XP_026407062.1	0	0.02650	0.97350	-0.65754

The upper 4 sequences shown in bold font are selected and tested NMCH sequences from *Eschscholzia californica* and *P. somniferum*. All other sequences are from *P. somniferum*. XP_026418925.1 is annotated as "(S)-*N*-methylcoclaurine 3'-hydroxylase isozyme 1", CYP80B1 and CYP80B3 in public databases. EcNMCH-Y202H contains an artificial binding pocket substitution to more closely resemble PsNMCH. PsNMCH-H203Y contains an artificial binding pocket substitution to more closely resemble EcNMCH. Abbreviations: NMCH - *N*-methylcoclaurine hydroxylase, NMCH-I1 - NMCH isozyme 1, G8HL - geraniol 8-hydroxylase-like, F3ML - flavonoid 3-monooxygenase-like, DHP6AML - 3,9-dihydroxypterocarpan 6A-monooxygenase-like, ECODL - 7-ethoxycoumarin *O*-deethylase-like, FSL - ferruginol synthase-like, PSOL - premnaspirodiene oxygenase-like, F35H1L - flavonoid 3,5-hydroxylase 1-like. All "C5167" sequences are annotated as "hypothetical protein".

Supplementary Table 5 | High-dimensional SVM-based prediction of *P. somniferum* CPR sequences

CPR prediction					
Annotation	Accession	Predict	Positive P	Negative P	Decision
NADPH-dependent diflavin oxidoreductase 1-like isoform X2	XP_026408903.1	1	0.55866	0.44134	0.04981
NADPH-dependent diflavin oxidoreductase 1-like isoform X1	XP_026408902.1	1	0.57537	0.42463	0.06423
**NADPH--cytochrome P450 reductase-like**	**XP_026404029.1**	**1**	**1.00000**	**0.00000**	**1.78640**
NADPH--cytochrome P450 reductase-like	XP_026394436.1	1	0.99736	0.00264	1.26978
NADPH--cytochrome P450 reductase-like	XP_026457702.1	1	1.00000	0.00000	1.76225
NADPH-dependent diflavin oxidoreductase 1-like isoform X2	XP_026439394.1	0	0.17645	0.82355	-0.33032
NADPH-dependent diflavin oxidoreductase 1-like isoform X1	XP_026439393.1	0	0.38367	0.61633	-0.10294
identical to XP_026404029.1	RZC71104.1	1	1.00000	0.00000	1.78640
hypothetical protein C5167_010877	RZC67195.1	1	0.99999	0.00001	1.35538
identical to XP_026404029.1	RZC56438.1	1	1.00000	0.00000	1.76225
NADPH:ferrihemoprotein oxidoreductase	AAC05021.1	1	0.99668	0.00332	1.22024

AAC05021.1 (NADPH:ferrihemoprotein oxidoreductase) was characterized as a cytochrome P450 reductase (CPR) in are previous report[32]. XP_026404029.1 (NADPH--cytochrome P450 reductase-like, abbreviated as PsCPR-L) is characterized in the current study and shown in bold font. High scoring sequence XP_026457702 is 99% identical to the selected sequence XP_026404029.1.

Supplementary Table 6 | High-dimensional SVM-based prediction of *P. somniferum* CYP450 sequences with potential tyrosine 3-monooxygenase activity

**CYP450 3-monooxygenase prediction**							
Annotation	Accession	CYP76AD Positive P	CYP76AD Negative P	CYP76AD Decision	Combined Positive P	Combined Negative P	Combined Decision
NMCH-I1	XP_026418925.1	0.09967	0.90033	-0.05708	0.55802	0.44198	-0.02370
NMCH, partial	ACM44071.1	0.00022	0.99978	-1.11956	0.00064	0.99936	-1.23916
NMCH-I1	Q9SP06.1	0.09113	0.90887	-0.07404	0.42287	0.57713	-0.11096
NMCH, partial	AAF61400.1	0.13999	0.86001	0.00891	0.41549	0.58451	-0.11588
NMCH-I1-like	XP_026389058.1	0.09832	0.90168	-0.05967	0.74931	0.25069	0.11441
C5167_025060	RZC63317.1	0.00022	0.99978	-1.12361	0.00021	0.99979	-1.41507
NMCH-like	XP_026431349.1	0.06249	0.93751	-0.14394	0.36335	0.63665	-0.15206
NMCH-like	I3V6B1.1	0.01272	0.98728	-0.42737	0.17580	0.82420	-0.30849
G8HL	XP_026380180.1	0.92852	0.07148	0.75836	0.96596	0.03404	0.47483
G8HL	XP_026411599.1	0.98067	0.01933	0.99184	0.95120	0.04880	0.41461
G8HL	XP_026380181.1	0.93378	0.06622	0.77243	0.92894	0.07106	0.35052
**G8HL**	**XP_026409442.1**	**0.99267**	**0.00733**	**1.16005**	**0.99652**	**0.00348**	**0.84531**
**C5167_048514**	**RZC73039.1**	**0.98333**	**0.01667**	**1.01765**	**0.80773**	**0.19227**	**0.16890**
G8HL	XP_026379063.1	0.71280	0.28720	0.47530	0.43382	0.56618	-0.10373
G8HL	XP_026380179.1	0.91344	0.08656	0.72276	0.49424	0.50576	-0.06468
C5167_004855	RZC57552.1	0.96449	0.03551	0.88480	0.00090	0.99910	-1.18485
G8HL	XP_026409440.1	0.87491	0.12509	0.65243	0.81581	0.18419	0.17735
G8HL	XP_026409437.1	0.78802	0.21198	0.54443	0.61490	0.38510	0.01298
G8HL	XP_026379135.1	0.95001	0.04999	0.82356	0.88381	0.11619	0.26380
G8HL	XP_026377890.1	0.56616	0.43384	0.36514	0.72476	0.27524	0.09411
CYP71A1-like	XP_026430038.1	0.05401	0.94599	-0.17769	0.41167	0.58833	-0.11844
C5167_026704	RZC86034.1	0.10892	0.89108	-0.04013	0.35503	0.64497	-0.15814
F3ML	XP_026441287.1	0.09569	0.90431	-0.06482	0.53842	0.46158	-0.03633
C5167_027831	RZC91763.1	0.04840	0.95160	-0.19688	0.20276	0.79724	-0.28031
CYP76A2-like	XP_026447672.1	0.07126	0.92874	-0.11985	0.84778	0.15222	0.21398
CYP76A2-like	XP_026447278.1	0.22551	0.77449	0.10847	0.68926	0.31074	0.06663
F3ML	XP_026381164.1	0.11624	0.88376	-0.02757	0.14318	0.85682	-0.34759
C5167_005813	RZC58514.1	0.02224	0.97776	-0.33146	0.01744	0.98256	-0.70935
CYP450	AFK73718.1	0.03506	0.96494	-0.25296	0.08685	0.91315	-0.43788
CYP76A2-like	XP_026450500.1	0.19700	0.80300	0.07913	0.85102	0.14898	0.21802
C5167_002759	RZC76181.1	0.02624	0.97376	-0.30296	0.04881	0.95119	-0.54300
DHP6AML	XP_026442746.1	0.16589	0.83411	0.04321	0.10784	0.89216	-0.39948
G8HL	XP_026411431.1	0.02026	0.97974	-0.34744	0.22728	0.77272	-0.25702
F3ML	XP_026427767.1	0.05762	0.94238	-0.16630	0.04139	0.95861	-0.56990
F3ML	XP_026418653.1	0.00702	0.99298	-0.52926	0.02579	0.97421	-0.64640
CYP736A12-like	XP_026430405.1	0.14287	0.85713	0.01297	0.08246	0.91754	-0.44695
CYP736A12-like	XP_026437612.1	0.33746	0.66254	0.20181	0.11411	0.88589	-0.38928
CYP76A2-like	XP_026437513.1	0.07915	0.92085	-0.10041	0.66892	0.33108	0.05168
CYP76A1-like	XP_026453156.1	0.36775	0.63225	0.22574	0.90913	0.09087	0.30766
CYP76A1-like	XP_026451658.1	0.13538	0.86462	0.00227	0.61347	0.38653	0.01206
G8HL	XP_026380438.1	0.00793	0.99207	-0.50840	0.21455	0.78545	-0.26887
G8HL	XP_026380437.1	0.00957	0.99043	-0.47618	0.28162	0.71838	-0.21099
G8HL	XP_026380436.1	0.00570	0.99430	-0.56473	0.11483	0.88517	-0.38814
C5167_048523, partial	RZC73046.1	0.69655	0.30345	0.46193	0.82713	0.17287	0.18970
CYP736A12-like	XP_026459019.1	0.00287	0.99713	-0.68233	0.00176	0.99824	-1.07710
ECODL	XP_026408386.1	0.00881	0.99119	-0.49036	0.15940	0.84060	-0.32734
G8HL	XP_026396971.1	0.23408	0.76592	0.11675	0.34545	0.65455	-0.16525
CYP736A12-like	XP_026388372.1	0.14401	0.85599	0.01455	0.03709	0.96291	-0.58770
CYP76A2-like	XP_026451160.1	0.11039	0.88961	-0.03755	0.76789	0.23211	0.13067
FSL isoform X1	XP_026380444.1	0.00344	0.99656	-0.65121	0.02518	0.97482	-0.65026
CYP76A1-like	XP_026399590.1	0.00757	0.99243	-0.51627	0.42563	0.57437	-0.10913
C5167_033939	RZC70799.1	0.00441	0.99559	-0.60872	0.25109	0.74891	-0.23604
CYP450	AFK73714.1	0.19894	0.80106	0.08123	0.54130	0.45870	-0.03448
G8HL	XP_026393993.1	0.18141	0.81859	0.06173	0.19336	0.80664	-0.28979
C5167_013475	RZC54568.1	0.00096	0.99904	-0.87047	0.00065	0.99935	-1.23745
CYP76C4-like	XP_026430487.1	0.00112	0.99888	-0.84331	0.03096	0.96904	-0.61692
CYP76C4-like	XP_026430486.1	0.00110	0.99890	-0.84627	0.02139	0.97861	-0.67653
CYP76A2-like	XP_026429624.1	0.57974	0.42026	0.37451	0.52533	0.47467	-0.04474
CYP736A12-like	XP_026460423.1	0.00848	0.99152	-0.49691	0.00211	0.99789	-1.04827
C5167_044785	RZC90154.1	0.03451	0.96549	-0.25566	0.09578	0.90422	-0.42063
CYP71A9-like	XP_026400228.1	0.03709	0.96291	-0.24318	0.05424	0.94576	-0.52569

CYP76C4-like	XP_026458250.1	0.00088	0.99912	-0.88525	0.01859	0.98141	-0.69913
C5167_015732	RZC56884.1	0.00075	0.99925	-0.91245	0.01943	0.98057	-0.69198
C5167_019753	RZC51326.1	0.53999	0.46001	0.34711	0.80717	0.19283	0.16832
CYP736A12-like	XP_026437453.1	0.14329	0.85671	0.01356	0.19329	0.80671	-0.28986
CYP71A9-like	XP_026444276.1	0.01737	0.98263	-0.37387	0.04853	0.95147	-0.54394
C5167_027648	RZC91585.1	0.20970	0.79030	0.09257	0.39769	0.60231	-0.12791
CYP71A9-like	XP_026378190.1	0.00183	0.99817	-0.75893	0.00754	0.99246	-0.84397
CYP736A12-like	XP_026388374.1	0.01802	0.98198	-0.36763	0.01057	0.98943	-0.78970
CYP71D8-like	XP_026451198.1	0.16519	0.83481	0.04234	0.53959	0.46041	-0.03557
CYP71A1-like	XP_026447646.1	0.08686	0.91314	-0.08305	0.08434	0.91566	-0.44301
CYP71A1-like isoform X1	XP_026380212.1	0.07484	0.92516	-0.11079	0.01135	0.98865	-0.77828
CYP736A12-like	XP_026460422.1	0.00120	0.99880	-0.83100	0.00092	0.99908	-1.18133
C5167_026541	RZC85882.1	0.28197	0.71803	0.15967	0.66588	0.33412	0.04949
C5167_005175	RZC57870.1	0.00000	1.00000	-1.92678	0.00022	0.99978	-1.40738
CYP71D8-like	XP_026392887.1	0.25363	0.74637	0.13491	0.56755	0.43245	-0.01755
C5167_009556	RZC65863.1	0.84695	0.15305	0.61242	0.01363	0.98637	-0.74897
**CYP450**	**AFK73720.1**	**0.11097**	**0.88903**	**-0.03653**	**0.99336**	**0.00664**	**0.74116**
CYP71A1-like	XP_026377587.1	0.18209	0.81791	0.06251	0.00616	0.99384	-0.87640
CYP76A2-like	XP_026443290.1	0.25540	0.74460	0.13651	0.36852	0.63148	-0.14834
CYP71A9-like	XP_026458455.1	0.05773	0.94227	-0.16597	0.16923	0.83077	-0.31587
CYP76A2-like	XP_026440631.1	0.04169	0.95831	-0.22288	0.12047	0.87953	-0.37945
ECODL	XP_026410296.1	0.06196	0.93804	-0.15350	0.56717	0.43283	-0.01780
**CYP98A2-like**	**XP_026458081.1**	**0.13579**	**0.86421**	**0.00286**	**0.99354**	**0.00646**	**0.74561**
**CYP98A2-like**	**XP_026403623.1**	**0.21957**	**0.78043**	**0.10259**	**0.99561**	**0.00439**	**0.80782**
CYP71D8-like	XP_026407054.1	0.17562	0.82438	0.05497	0.59168	0.40832	-0.00197
PSOL	XP_026392886.1	0.14410	0.85590	0.01468	0.57198	0.42802	-0.01469
CYP76A2-like	XP_026440487.1	0.51208	0.48792	0.32796	0.81885	0.18115	0.18061
CYP76A2-like	XP_026429558.1	0.40783	0.59217	0.25553	0.11780	0.88220	-0.38352
FSL	XP_026380449.1	0.01318	0.98682	-0.42127	0.01209	0.98791	-0.76818
CYP76AD1-like	XP_026380446.1	0.00284	0.99716	-0.68406	0.00734	0.99266	-0.84827
F3ML	XP_026380466.1	0.11481	0.88519	-0.02997	0.06315	0.93685	-0.49302
F3ML	XP_026410457.1	0.17611	0.82389	0.05555	0.05106	0.94894	-0.53563
CYP71A1-like	XP_026409602.1	0.05350	0.94650	-0.17933	0.00466	0.99534	-0.92099
**F35H1L**	**XP_026378021.1**	**0.42572**	**0.57428**	**0.26830**	**0.92543**	**0.07457**	**0.34218**
C5167_036448	RZC43501.1	0.12187	0.87813	-0.01838	0.65628	0.34372	0.04262
CYP71D9-like	XP_026407073.1	0.27543	0.72457	0.15410	0.63612	0.36388	0.02655
CYP76A2-like	XP_026429642.1	0.71282	0.28718	0.47532	0.20886	0.79114	-0.27434
C5167_026557	RZC85878.1	0.64566	0.35434	0.42240	0.02916	0.97084	-0.62662
PSOL	XP_026407062.1	0.01348	0.98652	-0.41736	0.16748	0.83252	-0.31787

High scoring sequences XP_026409442.1, RZC73039.1, AFK73720.1, XP_026458081.1, XP_026403623.1, XP_026378021.1 are shown in bold font. *P. somniferum* sequence abbreviations: NMCH - *N*-methylcoclaurine 3-hydroxylase, NMCH-I1 - NMCH isozyme 1, G8HL - geraniol 8-hydroxylase-like, F3ML - flavonoid 3-monooxygenase-like, DHP6AML - 3,9-dihydroxypterocarpan 6A-monooxygenase-like, ECODL - 7-ethoxycoumarin *O*-deethylase-like, FSL - ferruginol synthase-like, PSOL - premnaspirodiene oxygenase-like, F35H1L - flavonoid 3,5-hydroxylase 1-like. All C5167 sequences are annotated as "hypothetical protein". High scoring sequence RZC73039.1 (C5167_048514) shares homology with selected sequence XP_026409442.1 (G8HL). High scoring sequences AFK73720.1 (CYP450) and XP_026458081.1 (CYP98A2-like) share homology with selected sequence XP_026403623.1 (CYP98A2-like).

Supplementary Table 7 | List of plasmids used in this study

Plasmids	Description	Reference
pTrcHis2B	AmpR, empty expression plasmid, trc promoter system, pBR322 origin (4.4 kb)	Invitrogen
pTrc-DHPAAS	pTrcHis2B derivative, containing *DHPAAS* under trc promoter (5.9 kb)	*7*
pTrc-DHPAAS-S	pTrcHis2B derivative, containing *DHPAAS-N192H* under trc promoter (5.9 kb)	*7*
pTrc-DHPAAS-D	pTrcHis2B derivative, containing *DHPAAS-F79Y-Y80F* under trc promoter (5.9 kb)	*7*
pTrc-DHPAAS-T	pTrcHis2B derivative, containing *DHPAAS-F79Y-Y80F-N192H* under trc promoter (5.9 kb)	*7*
pBAD-DEST49	AmpR, empty expression plasmid, araBAD promote system, pUC origin (6.2 kb)	Invitrogen
pBAD-PsPDC1	pBAD-DEST49 derivative, containing *PsPDC1* under araBAD promoter (6.3 kb)	This study
pBAD-PsPDC2	pBAD-DEST49 derivative, containing *PsPDC2* under araBAD promoter (5.9 kb)	This study
pBAD-Ps2CHLL	pBAD-DEST49 derivative, containing *Ps2CHLL* under araBAD promoter (6.3 kb)	This study
pBAD-PsPDC1-His	pBAD-DEST49 derivative, containing His-tag fused *PsPDC1* under araBAD promoter (6.3 kb)	This study
pBAD-PsPDC2-His	pBAD-DEST49 derivative, containing His-tag fused *PsPDC2* under araBAD promoter (5.9 kb)	This study
pBAD-Ps2CHLL-His	pBAD-DEST49 derivative, containing His-tag fused *Ps2CHLL* under araBAD promoter (6.3 kb)	This study
pTXB1	AmpR, empty expression plasmid, T7 promoter system, pBR322 origin (6.7 kb)	NEB
pTXB1-PsTyDC1	pTXB1 derivative, containing *PsTyDC1* under T7 promoter (8.2 kb)	This study
pTYB21	AmpR, empty expression plasmid, T7 promoter system, pBR322 origin (7.5 kb)	NEB
pTYB21-PsTyDC1	pTYB21 derivative, containing *PsTyDC1* under T7 promoter (9.0 kb)	This study
pTYB21-PsTyDC6	pTYB21 derivative, containing *PsTyDC6* under T7 promoter (9.0 kb)	This study
pTYB21-TrcPsPDC1-IX1	pTYB21 derivative, containing N-terminal membrane domain truncated *PsPDC1-IX1* under T7 promoter (9.0 kb)	This study
pTYB21-TrcPsPDC1-IX1	pTYB21 derivative, containing N-terminal membrane domain truncated *PsPDC1-IX1* under T7 promoter with tandem lac repressor binding sites (9.3 kb)	This study
pTYB21-PsPDC1	pTYB21 derivative, containing *PsPDC1* under T7 promoter (9.3 kb)	This study
pTYB21-PsPDC2	pTYB21 derivative, containing *PsPDC2* under T7 promoter (8.9 kb)	This study
pTYB21-Ps2CHLL	pTYB21 derivative, containing *Ps2CHLL* under T7 promoter (9.2 kb)	This study
pTYB21-PpDDC-S	pTYB21 derivative, containing *PpDDC-H181L* under T7 promoter (8.9 kb)	This study
pET23a	AmpR, empty expression plasmid, T7 promoter system, pBR322 origin (3.7 kb)	Novagen
pET23a-EcHpaBC	pET23a derivative, containing *EcHpaB* and *EcHpaC* under T7 promoters (5.8 kb)	This study
pET23a-3PsMTs	pET23a derivative, containing optimized *Ps6OMT PsCNMT* and *Cj4OMT* under T7 promoters (9.2 kb)	*14*
pE-SUMO Kan	KmR, empty expression plasmid, T7 promoter system, pBR322 origin (5.6 kb)	LifeSensors
pE-DHPAAS	pE-SUMO derivative, containing *DHPAAS* under T7 promoter (7.1 kb)	7
pE-DHPAAS-S	pE-SUMO derivative, containing *DHPAAS-N192H* under T7 promoter (7.1 kb)	*7*
pE-DHPAAS-D	pE-SUMO derivative, containing *DHPAAS-F79Y-Y80F* under T7 promoter (7.1 kb)	*7*
pE-DHPAAS-T	pE-SUMO derivative, containing *DHPAAS-F79Y-Y80F-N192H* under T7 promoter (7.1 kb)	*7*
pACYC184	CmR, empty expression plasmid, p15A origin (4.3 kb)	Nippon Gene
pACYC-3CjMTs	pACYC184 derivative, containing *Cj6OMT*, *CjCNMT* and *Cj4OMT* under T7 promoters (7.4 kb)	*13*
pACYC-3CjMTs-PpDDC	pACYC184 derivative, containing *Cj6OMT*, *CjCNMT*, *Cj4OMT* and *PpDDC* under T7 promoters (9.0 kb)	*13*
pACYC-3CjMTs-PpDDC-S	pACYC184 derivative, containing *Cj6OMT*, *CjCNMT*, *Cj4OMT* and *PpDDC-H181L* under T7 promoters (9.0 kb)	This study
pACYC-3CjMTs-PpDDC-G344S	pACYC184 derivative, containing *Cj6OMT*, *CjCNMT*, *Cj4OMT* and *PpDDC-G344S* under T7 promoters (9.0 kb)	This study
pACYC-3CjMTs-PpDDC-G344I	pACYC184 derivative, containing *Cj6OMT*, *CjCNMT*, *Cj4OMT* and *PpDDC-G344I* under T7 promoters (9.0 kb)	This study
pACYC-3CjMTs-ppDDC-T	pACYC184 derivative, containing *Cj6OMT*, *CjCNMT*, *Cj4OMT* and *PpDDC-Y79F-F80Y-H181N* under T7 promoters (9.0 kb)	This study
pACYC-3CjMTs-PsNMCH	pACYC184 derivative, containing *Cj6OMT*, *CjCNMT*, *Cj4OMT* and truncated *PsNMCH* under T7 promoters (9.0 kb)	This study
pCDFDuet-1	SmR, empty expression plasmid, T7 promoter system, CloDF13 origin (3.8 kb)	Novagen
pCDFD-TfNCS	pCDFDuet-1 derivative, containing *TfNCS* under T7 promoter (4.3 kb)	This study
pCDFD-TfNCS-PsTyDC1	pCDFDuet-1 derivative, containing *TfNCS* and *PsTyDC1* under T7 promoters (5.8 kb)	This study
pCDFD-TfNCS-PsTyDC1-S	pCDFDuet-1 derivative, containing *TfNCS* and *PsTyDC1-L205H* under T7 promoters (5.8 kb)	This study
pCDFD-TfNCS-PsTyDC1-T	pCDFDuet-1 derivative, containing *TfNCS* and *PsTyDC1-Y98F-F99Y-L205N* under T7 promoters (5.8 kb)	This study
pCDFD-PsONCS3	pCDFDuet-1 derivative, containing *PsONCS3* under T7 promoter (5.8 kb)	This study
pCDFD-PsONCS3-PsTyDC1	pCDFDuet-1 derivative, containing *PsONCS3* and *PsTyDC1* under T7 promoters (7.2 kb)	This study
pCDFD-PsONCS3-	pCDFDuet-1 derivative, containing *PsONCS3* and *PsTyDC1-L205H* under T7 promoters (7.2 kb)	This study

PsTyDC1-S		
pCDFD-PsONCS3-PsTyDC1-T	pCDFDuet-1 derivative, containing *PsONCS3* and *PsTyDC1-Y98F-F99Y-L205N* under T7 promoters (7.2 kb)	This study
pCDFD-CjNCS-PpDDC-S	pCDFDuet-1 derivative, containing *CjNCS* and *PpDDC-H181L* under T7 promoters (6.2 kb)	This study
pCDFD-CjNCS-PpDDC-D	pCDFDuet-1 derivative, containing *CjNCS* and *PpDDC-H181L-G344S* under T7 promoters (6.2 kb)	This study
pCDFD-CjNCS-PpDDC-T	pCDFDuet-1 derivative, containing *CjNCS* and *PpDDC-Y79F-F80Y-H181N* under T7 promoters (6.2 kb)	This study
pCDFD-CjNCS-PpDDC-Q	pCDFDuet-1 derivative, containing *CjNCS* and *PpDDC-Y79F-F80Y-H181N-G344S* under T7 promoters (6.2 kb)	This study
pCDFD-CjNCS-ARO10	pCDFDuet-1 derivative, containing *CjNCS* and *ARO10* under T7 promoters (6.6 kb)	This study
pCOLAD-PsNMCH-AtATR2	pCOLADuet-1 derivative, containing *PsNMCH* and *AtATR2* under T7 promoters (6.9 kb)	This study
pCOLAD-PsNMCH-H203Y-AtATR2	pCOLADuet-1 derivative, containing *PsNMCH-H203Y* and *AtATR2* under T7 promoters (6.9 kb)	This study
pCOLAD-PsNMCH-PsCPR-L	pCOLADuet-1 derivative, containing *PsNMCH* and *PsCPR-L* under T7 promoters (6.9 kb)	This study
pCOLAD-PsNMCH-H203Y-PsCPR-L	pCOLADuet-1 derivative, containing *PsNMCH-H203Y* and *PsCPR-L* under T7 promoters (6.9 kb)	This study
pCOLAD-EcNMCH-AtATR2	pCOLADuet-1 derivative, containing *EcNMCH* and *AtATR2* under T7 promoters (7.0 kb)	This study
pCOLAD-EcNMCH-Y202H-AtATR2	pCOLADuet-1 derivative, containing *EcNMCH-Y202H* and *AtATR2* under T7 promoters (7.0 kb)	This study
pCOLAD-EcNMCH-PsCPR-L	pCOLADuet-1 derivative, containing *EcNMCH* and *PsCPR-L* under T7 promoters (7.0 kb)	This study
pCOLAD-EcNMCH-Y202H-PsCPR-L	pCOLADuet-1 derivative, containing *EcNMCH-Y202H* and *PsCPR-L* under T7 promoters (7.0 kb)	This study

The following species abbreviations are used in gene names: *Bm* - *Bombyx mori*, *Ps* - *Papaver somniferum*, *Ec* - *Escherichia coli*, *Cj* - *Coptis japonica*, *Tf* - *Thalictrum flavum*, *Pp* - *Pseudomonas putida*, *At* - *Arabidopsis thaliana*, *Ec* – *Eschscholzia californica*.

Supplementary Table 8 | List of primers used for molecular cloning

Name	Nucleotide sequence (5'-3')
PsTyDC1-L205H Fw	GCTTCTGATCAAACCCACAGTGCACTACAGAAAGCTGCTC
PsTyDC1-L205H Rv	GAGCAGCTTTCTGTAGTGCACTGTGGGTTTGATCAGAAGC
PsTyDC1-Y98F-F99Y Fw	CTTTGCTTTTTATCCTTCTAGTGGTTCTATCGCTGGTTTCC
PsTyDC1-Y98F-F99Y Rv	GGAAACCAGCGATAGAACCACTAGAAGGATAAAAAGCAAAG
PsTyDC1-L205N Fw	GCTTCTGATCAAACCAACAGTGCACTACAGAAAGCTGCTC
PsTyDC1-L205N Rv	GAGCAGCTTTCTGTAGTGCACTGTTGGTTTGATCAGAAGC
PsTyDC1 SapI Gibson Fw	TGATCATCAGTTTTTGCTTGGATCTCAGGTTGTTGTACAGAACATGGGAAGTCTTCCAGCTAATAACT
PsTyDC1 BamHI Gibson Rv	GCCGGATCAACTAGTTATTTAATTACCTGCAGGGAATTCGTTAACAAACATCCTCACCTAGTGCACC
PsTyDC1 SEQ1	GCCAGATGCTTACGCTTCCC
PsTyDC1 SEQ2	ACCAGTGAATCAGAGTCTTTCACCC
pTYB21-Fw	GCACGTGAGTGCCGCGG
CjNCS NdeI Fw	ATGATGATGCATATGAGCAAGAATCTTACTGGTGTGGGA
CjNCS XhoI Rv	CATCATCATCTCGAGTTATAGTTTCATCTGATCCAATAAGCTCTTACCATCA
PsONCS3 SEQ1	GCGCGCGTAATATTCTGT
PsONCS3 SEQ2	GTAATCAATTTGCTCATCGCTT
PsPDC1 SapI Gibson Fw	TGATCATCAGTTTTTGCTTGGATCTCAGGTTGTTGTACAGAACATGGATTTCAAAGTTGGTTCTCTTGATAC
PsPDC1 BamHI Gibson Rv	GCCGGATCAACTAGTTATTTAATTACCTGCAGGGAATTCGTTACTGAGGATTTGGTGGTCGACTGT
PsPDC2 SapI Gibson Fw	TGATCATCAGTTTTTGCTTGGATCTCAGGTTGTTGTACAGAACATGAAAAAAGCAGGCTTCCTATTGG
PsPDC2 BamHI Gibson Rv	GCCGGATCAACTAGTTATTTAATTACCTGCAGGGAATTCGCTACTGAGGATTGGGTGGGCG
Ps2HCLL SapI Gibson Fw	TGATCATCAGTTTTTGCTTGGATCTCAGGTTGTTGTACAGAACATGGCAGAAATCGATAACCTAACC
Ps2HCLL BamHI Gibson Rv	GCCGGATCAACTAGTTATTTAATTACCTGCAGGGAATTCGTTAGTTCTTGTGCTGCATTCTCCCACT
TrcPsPDC1-IX1 SapI Fw	ATGATGATGATGGCTCTTCCAACATGGGAAGTTCTCAAATTGAACTTGGAACC
TrcPsPDC1-IX1 BamHI Rv	CATCATCATCTGGATCCCTACTGAGGATTGGGTGGGCG
PsPDC1 SEQ1	GGAAGATGCACATGAGCAAATCG
PsPDC1 SEQ2	CACCCGTTTCGGCAATCAC
PsPDC2 SEQ1	AGGTCTGATACCTGAACATCAC
PsPDC2 SEQ2	ACAGCTGTGTCTCCGCTC
Ps2HCLL SEQ1	CGGTCGTCCTGGGGC
Ps2HCLL SEQ2	GCAGGGCTTCCCTGTGCTA
TrcPsPDC1-IX1 SEQ1	CACTAGGGAGCCTGTTCCG
M13 Fw	GTAAAACGACGGCCAGT
M13 Rv	CAGGAAACAGCTATGAC
ARO10 SEQ1	TTGCACATTGTTGGTGTGGC
ARO10 SEQ2	TCATGATATACTTTATGATTTGGCCC
ARO10 SEQ3	GACATCAATGAAATTAATAATGGGC
ARO10 SEQ4	CTCACATCAATGGTGGCAACG
ARO10 NcoI Fw	TTTTGTTTAACTTTAAGAAGGAGATATACCATGGCACCTGTTACAATTGAAAAGTT
ARO10 NotI Rv	TTCGACTTAAGCATTATGCGGCCGCCTATTTTTTATTTCTTTTAAGTGCCGCTGC
ACYCDuetUP1 Primer	GGATCTCGACGCTCTCCCT
DuetDOWN1 Primer	GATTATGCGGCCGTGTACAA
DuetUP2 Primer	TTGTACACGGCCGCATAATC
T7 Terminator Primer	GCTAGTTATTGCTCAGCGG
PpDDC Gibson NcoI Fw	TGTAGAAATAATTTTGTTTAACTTTAATAAGGAGATATACATGACCCCCGAACAATTCCG
PpDDC Gibson BamHI Rv	GCAAGCTTGTCGACCTGCAGGCGCGCCGAGCTCGAATTCGGATCCTCAGCCCTTGATCACGT
PpDDC Gibson SapI Fw	TGATCATCAGTTTTTGCTTGGATCTCAGGTTGTTGTACAGAACATGACCCCCGAACAATTCC
PpDDC Gibson BamHI Rv	GCCGGATCAACTAGTTATTTAATTACCTGCAGGGAATTCGTCAGCCCTTGATCACGTCCTGC
PpDDC-Y79F-F80Y Fw	GCACCCGGACTTCTATGGCTTTTACCCTTCCAATGGCACCCTGTCC
PpDDC-Y79F-F80Y Rv	GGACAGGGTGCCATTGGAAGGGTAAAAGCCATAGAAGTCCGGGTGC
PpDDC-H181N Fw	CGTGTATGTCAGCGCCCACGCCAACAGCTCGGTGGACAAGGCTGCAC

PpDDC-H181N Rv	GTGCAGCCTTGTCCACCGAGCTGTTGGCGTGGGCGCTGACATACACG
PpDDC-H181L Fw	CGTGTATGTCAGCGCCCACGCCCTCAGCTCGGTGGACAAGGCTGCAC
PpDDC-H181L Rv	GTGCAGCCTTGTCCACCGAGCTGAGGGCGTGGGCGCTGACATACACG
PpDDC-G344S Fw	TGCGCGACTGGGGGATACCGCTGAGCCGTCGGTTCCGTGCGTTGAAG
PpDDC-G344S Rv	CTTCAACGCACGGAACCGACGGCTCAGCGGTATCCCCCAGTCGCGCA
PpDDC-G344I Fw	TGCGCGACTGGGGGATACCGCTGATCCGTCGGTTCCGTGCGTTGAAGC
PpDDC-G344I Rv	GCTTCAACGCACGGAACCGACGGATCAGCGGTATCCCCCAGTCGCGCA
PpDDC SEQ1	TGCCCGTGAACGCGCCA
PpDDC SEQ2	GTCGCGCAGGTTCTTCACCT
PsNMCH SEQ1	ACGCAGAAGATGATTGAAAGTCAAGC
PsNMCH SEQ2	GCTTGACTTTCAATCATCTTCTGCGT
PsNMCH SEQ3	CCAACCGCATATTGGCTGTTACT
PsNMCH NotI Fw	ATGATGGCGGCCGC
PsNMCH XhoI Rv	CATCATCTCGAGTTAATCCCGAGTTTTAGGA
PsNMCH Overlap Fw	TTTTGTTTAACTTTAAGAAGGAGATATACCATGGATTCAAGTCCTAAAGGTTTGCCACCA
T7 promotor element Overlap Rv	GGCAAACCTTTAGGACTTGAATCCATGGTATATCTCCTTCTTAAAGTTAAACAAAATTATTTCT
PsNMCH H203Y Fw	GTGGAAGTGTAGAAATGAAAGAATATCTATGGAGAATGCTGGAATTGGGG
PsNMCH H203Y Rv	CCCCAATTCCAGCATTCTCCATAGATATTCTTTCATTTCTACACTTCCAC
EcNMCH Y202H Fw	GTTCAGAGTTCAAGGAACATCTATGGAGGATGTTGGAATTGGGGAATTC
EcNMCH Y202H Rv	GAATTCCCCAATTCCAACATCCTCCATAGATGTTCCTTGAACTCTGAAC
EcNMCH SEQ1	TCCGCTCGTTACGTTTTTCAAAGTTTTCGAGTAAAAGGGCATGTAG
EcNMCH SEQ2	CGTGGGAGTAGCAATGGAGTTGGTGGGTGAAGTCTTAGGG
PsNMCH NcoI Fw	TTTTGTTTAACTTTAAGAAGGAGATATACCATGGATTCAAGTCCTAAAG
PsNMCH NotI Rv	CATCATCATGCGGCCGCTTAATCCCGAGTTTTAGGAACAATATACAGAGGTGG
PsNMCH Gibson Fw	TACCATGGGCAGCAGCCATCACCATCATCACCACAGCCAGATGGATTCAAGTCCTAAAGGTTTGCC
PsNMCH Gibson Rv	GTACAATACGATTACTTTCTGTTCGACTTAAGCATTATGCTTAATCCCGAGTTTTAGGAACAATATACAGAG

Supplementary Table 9 | Codon optimized gene sequences

**Optimized sequence of PsONCS3**
ATGCGCAAAGTGATTAAATACGATATGGAAGTGGCGGTTTCTGCTGACAGTGTCTGGGCGGTCTATTCATCGCC
AGATATTCCACGCTTGTTGCGTGACGTCTTACTGCCCGGCGTGTTTGAAAAATTAGACGTGATCGAAGGCAATG
GCGGCGTCGGCACCGTGCTGGATATTGTTTTCCCGCCAGGCGCGGTTCCGCGTTCTTATAAAGAGAAGTTCGTG
AATATTGATCGCGAAAAACGCCTGAAAGAGGTCATTATGATTGAAGGGGGTTATCTTGATATGGGCTGCACCTT
CTATCTGGACCGTATTCACGTTGTCGAAAAAACCAAAAGCAGTTGCGTCATTGAATCCAGTATTGTGTACGACG
CGAAAGAAGAATGCGCCGATGCGATGAGTAAACTGATTACTACCGAGCCGTTGAAATCGATGGCAGAAGTGAT
TAGCAATTATGTCATTCAAAAAGAGAGTTTTAGCGCGCGTAATATTCTGTCAAAACAAAGCGTGGTAAAAAAGG
AAATTCGCTATGACCTGGAGGTGCCGATTTCTGCGGATTCTATCTGGAGCGTATATAGCTGTCCGGACATCCCGC
GTCTGTTACGTGATGTGCTGTTGCCTGGCGTGTTCGAAAAGCTGGATGTGATCGAGGGCGATGGCGGCGTTGGT
ACCGTTCTGGACATTGTCTTCCCGCCGGGGGCGGTGCCGCGCAGCTACAAAGAGAAGTTTGTCAATATCGACCG
CGAGAAACGTCTCAAAGAAGTTATCATGATCGAAGGTGGCTACCTGGATATGGGCTGTACCTTTTACCTCGACC
GCATTCATGTGGTTGAAAAATCTCTGTCTTCCTGCGTGATTGAGAGCTCCATCGTTTATGAAGTGAAAGAAGAG
TATGTTGATGCCATGTCGAAACTGATCACCACGGAACCGCTGAAAAGCATGGCTGAAGTTATCAGCAACTATGT
GATTCAGCGTGAAAGCTTCAGCGCCCGCAATATCCTGAATAAAAATAGCCTGGTTAAGAAAGAGATTCGTTATG
ATCTGGAAGTCCCGACCTCTGCCGACAGCATCTGGTCGGTTTACAGCTGCCCGGATATCCCACGTTTATTACGCG
ACGTTTTGCTGCCGGGCGTATTCCAGAAACTCGATGTTATAGAAGGCAACGGCGGTGTCGGTACGGTACTGGAT
ATCGTCTTTCCACCGGGTGCGGTACCGCGCAGTTATAAAGAGAAATTCGTTAACATCAACCATGAAAAGCGTCT
GAAGGAGGTTATTATGATTGAAGGCGGTTACCTTGATATGGGGTGTACCAGCTATCTCGATCGCATCCATGTCG
TTGAGAAAACAAGCAAATCCTGTATTATTAAGAGCAGCGTTGTTTATGAGGTGAAGCAGGAATGTGTGGAAGC
GATGAGCAAATTGATTACCACCGAACCACTGAAATCAATGGCGGAAGTCATCAGTAACTACGCAATGAAACAG
CAGAGCGTCAGCGAACGTAACATTCCGAAAAAACAGTCGCTGCTGCGTAAAGAAATCACCTACGAAACCGAAG
TGCAGACCAGTGCTGATAGCATTTGGAACGTGTATTCCAGCCCGGATATTCCTCGCCTGCTGCGCGATGTCCTGC
TTCCCGGTGTTTTTGAAAAACTGGATGTAATTGCCGGTAACGGTGGTGTGGGCACGGTGTTGGATATCGCCTTTC
CGCTGGGCGCAGTGCGTCGCCGCTACAAAGAAAAATTTGTTAAAATTAACCACGAAAAGCGCTTAAAAGAAGT
GGTGATGATCGAAGGTGGCTATCTGGACATGGGTTGTACGTTTTATATGGATCGTATCCACGTATTTGAGAAAA
CGCCGAACAGCTGCGTTATTGAAAGTTCGATTATCACTAAACTTAAAAAAAGTATGCTGGTGAAATGGCTGAGT
TAA

**Optimized sequence of TfNCS**
ATGAAATTAATCCTGACCGGTCGCCCGTTTTTACATCATCAGGGCATCATCAACCAGGTGAGCACCGTCACCAA
AGTCATTCACCACGAACTGGAAGTGGCGGCCAGCGCCGATGATATCTGGACGGTTTACAGCTGGCCGGGTCTGG
CGAAACATCTGCCGGATCTGCTGCCTGGCGCGTTTGAAAAGCTGGAAATTATCGGCGATGGCGGCGTTGGCACC
ATCCTCGATATGACCTTTGTTCCGGGCGAATTCCCGCATGAATACAAAGAAAAATTTATTCTGGTTGATAACGA
ACATCGCCTGAAAAAAGTGCAGATGATTGAAGGCGGTTATCTGGATCTCGGCGTGACCTATTATATGGATACCA
TTCATGTGGTGCCGACGGGTAAAGATAGCTGCGTGATTAAATCGTCCACCGAATATCACGTTAAACCGGAATTT
GTGAAAATCGTTGAACCGCTGATCACCACCGGCCCGCTGGCAGCGATGGCGGATGCCATCAGCAAACTGGTGC
TGGAGCATAAAAGTAAAAGCAACAGCGATGAAATCGAAGCGGCGATTATTACCGTTTAA
