# Supplementary material for: Machine learning discovery of missing links that mediate alternative branches to plant alkaloids
Source: Nat Commun. 2022 Mar 16;13:1405. doi: 10.1038/s41467-022-28883-8 (PMC8927377; doi:10.1038/s41467-022-28883-8)
Supplement: Supplementary file 2 — Description of Additional Supplementary Files [file 41467_2022_28883_MOESM2_ESM.pdf]

Description of Additional Supplementary Files

File Name: Supplementary Data 1
Description: Training sequences and information for support vector machine (SVM) and random forests models to predict aromatic amino acid decarboxylase (AAAD)

File Name: Supplementary Data 2
Description: Training sequences and information for SVM and random forests models to predict aromatic acetaldehyde synthase (AAS)

File Name: Supplementary Data 3
Description: Training sequences and information for the combined SVM model to predict phenylpyruvate decarboxylase (PPDC)

File Name: Supplementary Data 4
Description: Training sequences and information for specific SVM and random forests models to predict PPDC

File Name: Supplementary Data 5
Description: Training sequences and information for the SVM model to predict *N*-methylcoclaurine 3-hydroxylase (NMCH)

File Name: Supplementary Data 6
Description: Training sequences and information for the SVM model to predict CYP450 reductase (CPR)

File Name: Supplementary Data 7
Description: Training sequences and information for the SVM model to predict CYP76AD

File Name: Supplementary Data 8
Description: Training sequences and information for the combined SVM model to predict CYP76AD, CYP98A3 and CYP199A2
