# Supplementary material for: Machine learning discovery of missing links that mediate alternative branches to plant alkaloids
Source: Nat Commun. 2022 Mar 16;13:1405. doi: 10.1038/s41467-022-28883-8 (PMC8927377; doi:10.1038/s41467-022-28883-8)
Supplement: Supplementary file 11 — Reporting Summary [file 41467_2022_28883_MOESM11_ESM.pdf]

nature research

Corresponding author(s): Christopher Vavricka, Michihiro Araki and Tomohisa Hasunuma

Last updated by author(s): February 13th, 2022

Reporting Summary

Nature Research wishes to improve the reproducibility of the work that we publish. This form provides structure for consistency and transparency in reporting. For further information on Nature Research policies, see our Editorial Policies and the Editorial Policy Checklist.

Statistics

For all statistical analyses, confirm that the following items are present in the figure legend, table legend, main text, or Methods section.

n/a	Confirmed	
☐	☒	The exact sample size (*n*) for each experimental group/condition, given as a discrete number and unit of measurement
☐	☒	A statement on whether measurements were taken from distinct samples or whether the same sample was measured repeatedly
☒	☐	The statistical test(s) used AND whether they are one- or two-sided<br>*Only common tests should be described solely by name; describe more complex techniques in the Methods section.*
☒	☐	A description of all covariates tested
☒	☐	A description of any assumptions or corrections, such as tests of normality and adjustment for multiple comparisons
☐	☒	A full description of the statistical parameters including central tendency (e.g. means) or other basic estimates (e.g. regression coefficient) AND variation (e.g. standard deviation) or associated estimates of uncertainty (e.g. confidence intervals)
☒	☐	For null hypothesis testing, the test statistic (e.g. *F*, *t*, *r*) with confidence intervals, effect sizes, degrees of freedom and *P* value noted<br>*Give P values as exact values whenever suitable.*
☒	☐	For Bayesian analysis, information on the choice of priors and Markov chain Monte Carlo settings
☒	☐	For hierarchical and complex designs, identification of the appropriate level for tests and full reporting of outcomes
☒	☐	Estimates of effect sizes (e.g. Cohen's *d*, Pearson's *r*), indicating how they were calculated

Our web collection on statistics for biologists contains articles on many of the points above.

Software and code

Policy information about availability of computer code

Data collection	GC-MS, LC-MS and CE-MS were operated with Shimadzu LabSolutions 2.72, Shimadzu LabSolutions LCMS 5.99 SP2 and Agilent MassHunter 10.1, respectively.
Data analysis	Data was analyzed using our custom code [https://github.com/nwatanbe/SVM_E_model], Python 3.7.4, scikit-learn 0.21.3, Shimadzu LabSolutions (above), Agilent MassHunter B.06.00, Prism 7 7.0d, ChemDraw 19.0.1.32, Modeller in Chimera 1.15, PyMOL 1.8.7.0, Molecular Operating Environment 2020.0901, NUS COOL (citation 61), and based on code from https://towardsdatascience.com/visualizing-support-vector-machine-decision-boundary-69e7591dacea.

For manuscripts utilizing custom algorithms or software that are central to the research but not yet described in published literature, software must be made available to editors and reviewers. We strongly encourage code deposition in a community repository (e.g. GitHub). See the Nature Research guidelines for submitting code & software for further information.

Data

Policy information about availability of data

All manuscripts must include a data availability statement. This statement should provide the following information, where applicable:

- Accession codes, unique identifiers, or web links for publicly available datasets
- A list of figures that have associated raw data
- A description of any restrictions on data availability

The following data availability statement is included: Data supporting the findings of this work are available within the paper and its Supplementary Information files. Training sequences and information for machine learning models are included as Supplementary Data files. A reporting summary for this Article is available as a Supplementary Information file. Source data for Figures 3c, 5d, 6b, 7a, 7b, 8a, 8b and 8c are provided as a Source Data file. All protein accessions used in this study are available from the National Center for Biotechnology Information (NCBI) database [https://www.ncbi.nlm.nih.gov]. Protein Data Bank (PDB) structures including

2Q5O and 2WVA are available from the PDB database [www.rcsb.org].

Field-specific reporting

Please select the one below that is the best fit for your research. If you are not sure, read the appropriate sections before making your selection.

[x] Life sciences [ ] Behavioural & social sciences [ ] Ecological, evolutionary & environmental sciences

For a reference copy of the document with all sections, see nature.com/documents/nr-reporting-summary-flat.pdf

Life sciences study design

All studies must disclose on these points even when the disclosure is negative.

Sample size	In most cases, sample size (n) represents the number of times an individual dilution, prepared from an individual sample, was analyzed. In the remaining cases, multiple dilutions were prepared from the same sample, and run independently. A sample size (n) of 3 was used for most samples to meet the journal standards. When screening a large number of samples that might not all be necessary for publication, sometimes a sample size (n) of 2 was used.
Data exclusions	Data was rarely excluded. However, in a few cases mass spectral data was excluded if the target peak was over-saturated, if the internal standard peak did not match that of other data, or if the compound peak for an unstable compound had degraded after a long delay before analysis.
Replication	Quantitative samples were run in replicates, and new enzymes were tested in 2 or more independent experiments. All attempts at replication were successful.
Randomization	Sample analysis order was random to lower bias for unstable compounds.
Blinding	For samples that were not blinded, it was possible that sample order could effect the signal intensity for some unstable compounds. To control for this, replicates were not started until the entire set of samples were finished running in the first round. For the enzyme assay in Fig. 3c, conditions were started in reverse order for one replicate to control for any possible effects. As a result, sample order had no effect on the study outcomes.

Reporting for specific materials, systems and methods

We require information from authors about some types of materials, experimental systems and methods used in many studies. Here, indicate whether each material, system or method listed is relevant to your study. If you are not sure if a list item applies to your research, read the appropriate section before selecting a response.

Materials & experimental systems

n/a	Involved in the study	
[x]	[ ]	Antibodies
[x]	[ ]	Eukaryotic cell lines
[x]	[ ]	Palaeontology and archaeology
[x]	[ ]	Animals and other organisms
[x]	[ ]	Human research participants
[x]	[ ]	Clinical data
[x]	[ ]	Dual use research of concern

Methods

n/a	Involved in the study	
[x]	[ ]	ChIP-seq
[x]	[ ]	Flow cytometry
[x]	[ ]	MRI-based neuroimaging
